# Scaling European Citizen Driven Transferable and Transformative Digital Health: Protocol for an Effectiveness-Implementation Hybrid Trial of a Digital Health Platform to Support Multimorbidity Self-Management

**DOI:** 10.2196/74989

**Published:** 2025-11-25

**Authors:** Julie Doyle, Séamus Harvey, Sara Polak, Myriam Sillevis Smitt, Jane Murphy, Áine Teahan, Jessica Ferreira Morais, Suzanne Smith, Orla Moran, Gordon Boyle, An Jacobs, Sarah Anne Tighe, John Dinsmore

**Affiliations:** 1 NetwellCASALA Dundalk Institute of Technology Dundalk Ireland; 2 imec-SMIT Vrije Universiteit Brussel Brussels Belgium; 3 Trinity Centre for Practice and Healthcare Innovation Trinity College Dublin Dublin Ireland; 4 Health Research Board Dublin Ireland

**Keywords:** multimorbidity, digital health, chronic disease, self-management, aging, older adults, effectiveness-implementation hybrid trial

## Abstract

**Background:**

Multimorbidity, the presence of 2 or more chronic conditions, is becoming increasingly prevalent worldwide, resulting in significant impacts on health care systems. For people with multimorbidity, self-management is challenging, requiring engagement in several tasks. Digital health platforms have been widely acknowledged as having the potential to enhance self-management practices for those with chronic conditions. However, limited longitudinal studies have explored the effectiveness of digital health platforms that support multimorbidity self-management or issues relating to their implementation and scalability in practice.

**Objective:**

The aim of this study is to determine the effectiveness and implementation of a digital health platform, ProACT, with support services including clinical triage and a care network (consisting of informal and formal caregivers and health care professionals) compared to the use of the platform alone and compared to standard care.

**Methods:**

An effectiveness-implementation type 1 hybrid study will be conducted across 3 European countries. A total of 720 older adults aged 65 years or older with multimorbidity (2 or more of the following: diabetes, a chronic respiratory disease, chronic heart failure, and chronic heart disease) will be recruited and randomized into 1 of 3 trial arms. Those in trial arm 1 will be invited to have up to 5 care network members participate with them, resulting in a maximum of 1500 care network participants. Effectiveness will be assessed through a 3-arm pragmatic randomized controlled trial, while implementation issues will be addressed via a process evaluation. Primary outcomes for participants with multimorbidity are quality of life and health care use, while secondary outcomes focus on the potential of the ProACT platform to support multimorbidity self-management (eg, self-efficacy, usability, engagement, and symptom stabilization). Primary outcomes for informal caregivers in the care network include caring burden, while secondary outcomes for all care network members include usability, engagement, satisfaction, and overall experiences with ProACT. Additional outcomes related to the process evaluation include the reach, uptake, and fidelity of implementation of ProACT and the way organizations implement and deliver ProACT; how they differ in this regard; and the factors underpinning these differences. A range of qualitative and quantitative data will be collected and analyzed to assess these outcomes.

**Results:**

Enrollment in the trial began in September 2022, and the trial is anticipated to end by March 2026. Trial outcomes will be submitted for publication in 2026.

**Conclusions:**

The generation of evidence-based support for the routine use of the ProACT platform in applied settings would represent considerable impact. With health care services under increasing strain and traditionally designed to support those with single morbidities, it is more important than ever to develop actionable insights and resources to empower persons with multimorbidity to self-manage their complex care needs at home, with support from their caregivers.

**Trial Registration:**

ISRCTN Registry ISRCTN34134007; https://www.isrctn.com/ISRCTN34134007

**International Registered Report Identifier (IRRID):**

DERR1-10.2196/74989

## Introduction

### Background

Approximately 50 million people in the European Union, including at least 60% of those aged 65 years and older, live with multiple chronic conditions or multimorbidity [1-3], which is typically defined as the presence of 2 or more chronic conditions in the same individual [4]. The older adult population of the European Union is also increasing; the share of those aged 80 years and older is projected to increase from 5.5% to 12.7% between 2017 and 2080 [5]. Given the link between multimorbidity and increased age [6-8], combined with rising health care expenditures and budgetary constraints, health care systems face considerable challenges in sustaining services to meet the growing demands for long-term health care provision. For instance, the European Union spends approximately US $ 816 billion per annum on chronic disease management, comprising 70% to 80% of total health care costs [9], with health care costs in general projected to rise further [10]. Health care systems need to evolve, and central to this is an understanding that multimorbidity is becoming the norm rather than an exception. At present, health care systems are designed to support those with 1 rather than multiple chronic conditions; they instead need to address the management of multiple conditions, where difficulties and conflicts in care and treatment can often arise [11-14].

For people with multimorbidity, services are often repetitive, inconvenient, and burdensome (eg, due to the need to attend multiple appointments), as well as inefficient, fragmented, and potentially unsafe (eg, people with multimorbidity may receive conflicting advice from different clinicians in relation to different conditions) [15-17]. Those with multimorbidity must also often take several medications, which can be difficult to keep track of, with some combinations potentially dangerous [18,19]. There is a need to improve best practices around the provision of well-coordinated person-centered care. One way to achieve this is to empower people with multimorbidity to use digital health technology to play an active role in the self-management of their health and well-being, supported by their care networks. A digital integrated care platform, designed with the complexities of multimorbidity in mind, may facilitate self-management at home. Such a platform may also facilitate greater coordination between people with multimorbidity and their care networks in relation to their health care; while overall, it may ensure that health care systems become more sustainable, “future-proofed,” and accessible to people with multimorbidity when necessary [20].

However, while older adults are increasingly open to using digital health technology, this technology type is still in its infancy and lacks widespread adoption [21]. Furthermore, although digital health technology has demonstrated potential to advance self-management and care for those with multimorbidity [22,23], research examining its efficacy is limited. Research into digital self-management of multimorbidity has typically explored design recommendations only [24]. Our previous work represents one of the few studies to test a digital health platform (ProACT) for multimorbidity self-management and care at a proof-of-concept level among people with multimorbidity and their care networks [24,25]. The quantitative and qualitative findings revealed that the ProACT platform was engaging and useful; the qualitative findings also revealed that it facilitated perceived improvements in participants’ health and well-being, self-management, and support [24,26,27].

### Study Aim and Objectives

The overall aim of the Scaling European Citizen Driven Transferable and Transformative Digital Health (SEURO) project is to evaluate key factors to prepare organizations, localities, and regions across the European Union to scale, sustain, and transfer people-centric digital integrated health and social care solutions for multiple disease management. To achieve this, an effectiveness-implementation hybrid (EIH) trial [28] will be carried out in Ireland, Belgium, and Sweden, with ProACT, with embedded process evaluations to understand the process for successful “real-world” implementation. Effectiveness will be assessed from the user perspective via perceived quality of life (QoL) and from the service perspective via health care use (HCU); implementation will be assessed using quantitative and qualitative data (eg, assessment of organization readiness, participants’ system use data, health care organizations’ administrative data, and interview data) collected as part of a process evaluation. Effectiveness will be assessed through a 3-arm pragmatic randomized controlled trial (p-RCT) in each trial site. Those in trial arm 1 will receive a personalized version of the ProACT platform, can invite people in their care network (eg, informal and formal caregivers and health care professionals) to participate with them, and will have access to clinical triage support. Those in trial arm 2 will receive a basic, nonpersonalized ProACT CareApp with no access to the care network or clinical triage, while those in trial arm 3 will continue to receive standard care.

The objectives of this trial are (1) to evaluate the impact of the ProACT platform on people with multimorbidity’s QoL and HCU (ie, the trial primary outcomes); participants in each trial arm will be compared on these variables; (2) to evaluate the cost-effectiveness of the personalized ProACT CareApp with care network and triage support in comparison to the nonpersonalized ProACT CareApp and control group; (3) to explore via a mixed methods approach (analysis of system and questionnaire data and interviews with participants) the potential impact of ProACT on multimorbidity self-management; (4) to evaluate the impact of the ProACT platform on informal caregivers’ caring burden; and (5) to evaluate participants’ (people with multimorbidity and care networks) overall experiences with the ProACT platform in the management of multimorbidity. Data collected from participants in trial arms 1 and 2 will also be compared. Furthermore, to explore the impact of contextual factors (eg, participants’ conditions, age, and socioeconomic status and care network participants’ motivation to take part in the trial) on fidelity of use and use outcomes (eg, reach, uptake, and acceptability); (6) to understand how ProACT is implemented and delivered in health care settings and to identify systematic differences and variation in delivery; (7) to evaluate via the process evaluation organizational readiness to transfer and optimize a digital health solution as part of the care delivery; and (8) to evaluate whether trial outcomes can inform future analysis of the impact of digital solution implementation in practice. This protocol adheres to the SPIRIT (Standard Protocol Items: Recommendations for Interventional Trials) guidelines for clinical trial reporting (Multimedia Appendix 1) [29].

### The ProACT Digital Health Platform

#### Overview

In total, 166 key stakeholders (ie, people with multimorbidity and care network members) were involved in the design and development process of the first version of the ProACT digital health platform, as part of the ProACT Horizon 2020 project [24]. This extensive requirement-gathering process included semistructured interviews, focus groups, co-design workshops, and usability testing, aiming to ensure that the platform’s user interface and content were understandable, relevant, and compatible with the daily lives of older individuals with varying cognitive capacities, health literacy, and digital literacy. Furthermore, during the ProACT project, the ProACT platform was tested and refined at a proof-of-concept level with 120 older adults with multimorbidity in Ireland and Belgium during a 12-month period, with promising outcomes observed, particularly in user engagement [24,27]. Extensive feedback was also collected from users on usability during the ProACT trial, resulting in updates and refinements being made to the platform, primarily in terms of aesthetics and navigation.

The ProACT digital health platform that will be deployed in trial arm 1 of the SEURO EIH trial is composed of the following elements.

#### Measuring Devices

These include “off-the-shelf” devices that are used to collect health and well-being data from people with multimorbidity in their homes (eg, blood glucose, blood pressure, pulse oximetry, heart rate, weight, sleep, and activity measures).

#### The ProACT CareApp

**Figure 1:**
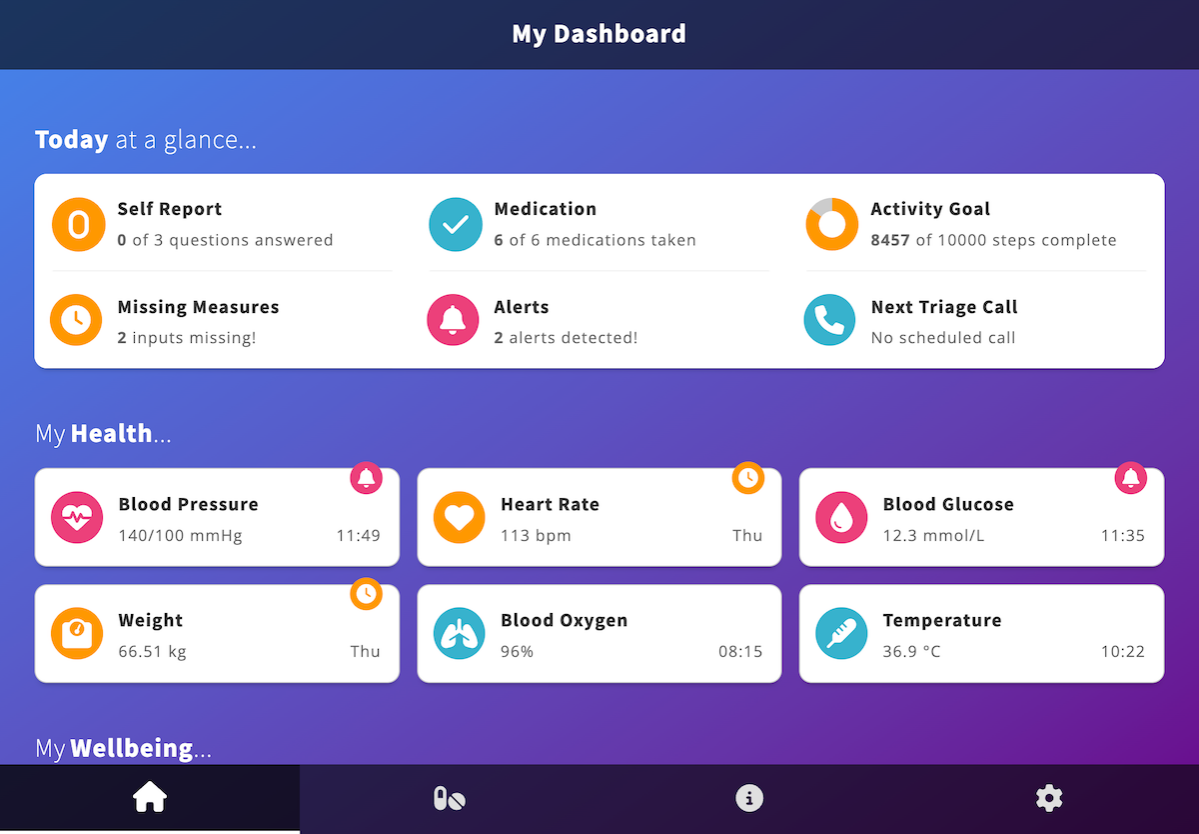
The ProACT CareApp dashboard showing an overview of today’s status, and sections showing data related to health parameters (My Health), with similar presentations of data for well-being parameters (My Wellbeing) and activity parameters (My Activity) further down the page.

**Figure 2:**
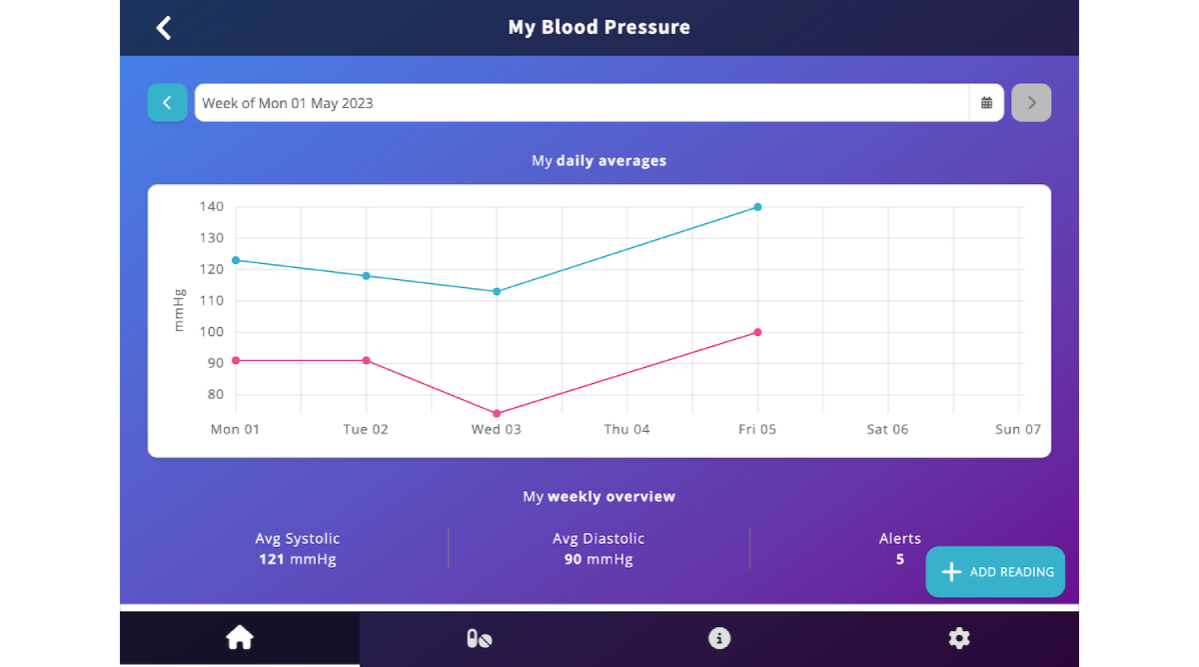
Tapping on individual health, well-being, or activity cards on the dashboard brings the user to a more detailed view of their data. The figure shows an example of weekly blood pressure readings.

**Figure 3:**
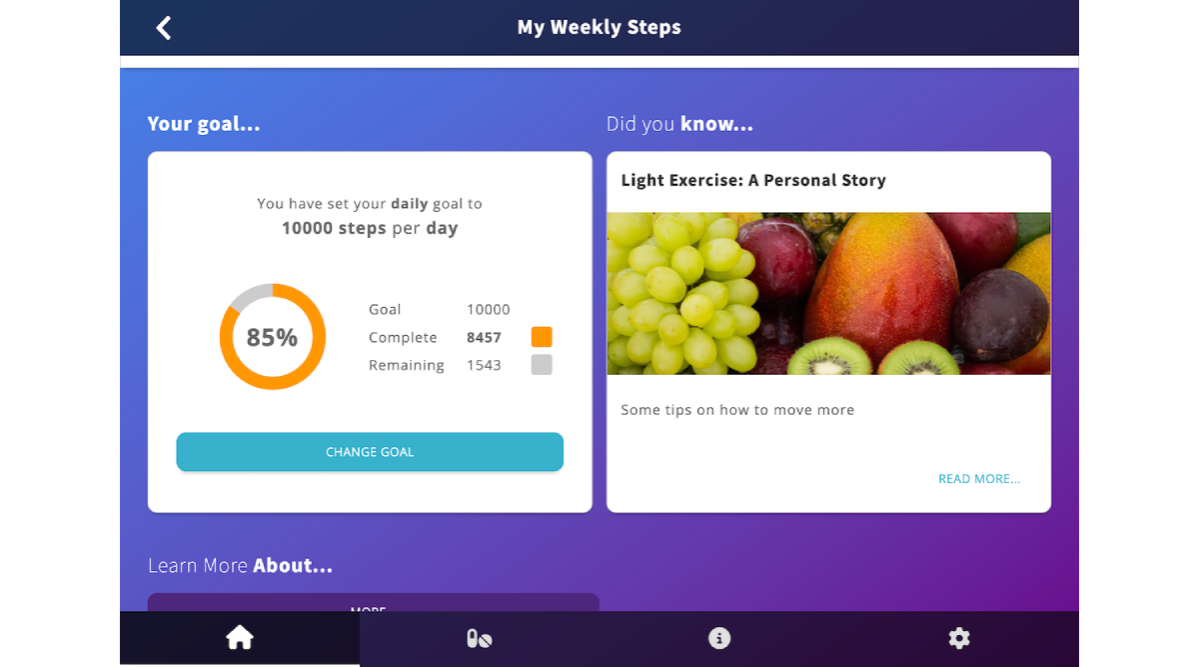
Users can set an activity or a weight goal and track their progress. A piece of contextually relevant education is also displayed on this screen.

**Figure 4:**
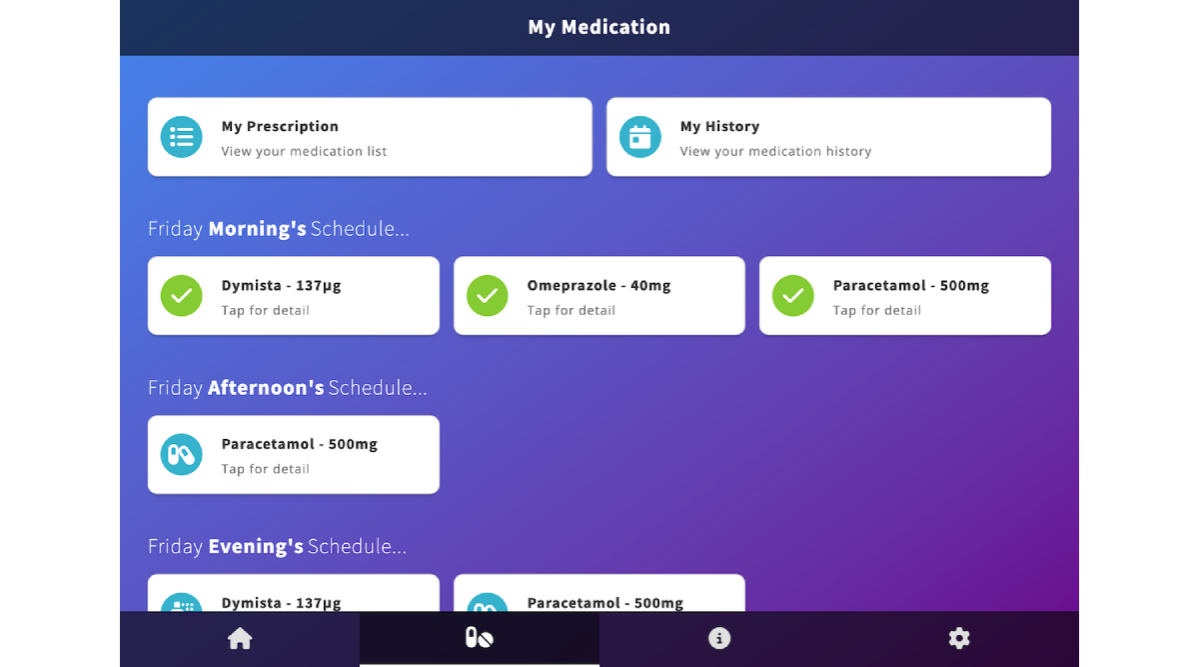
The medication management section (My Medication) of the CareApp, where users can enter or add to their prescription, view their schedule, and track their intake by tapping on a medication and indicating that they have taken it today.

**Figure 5:**
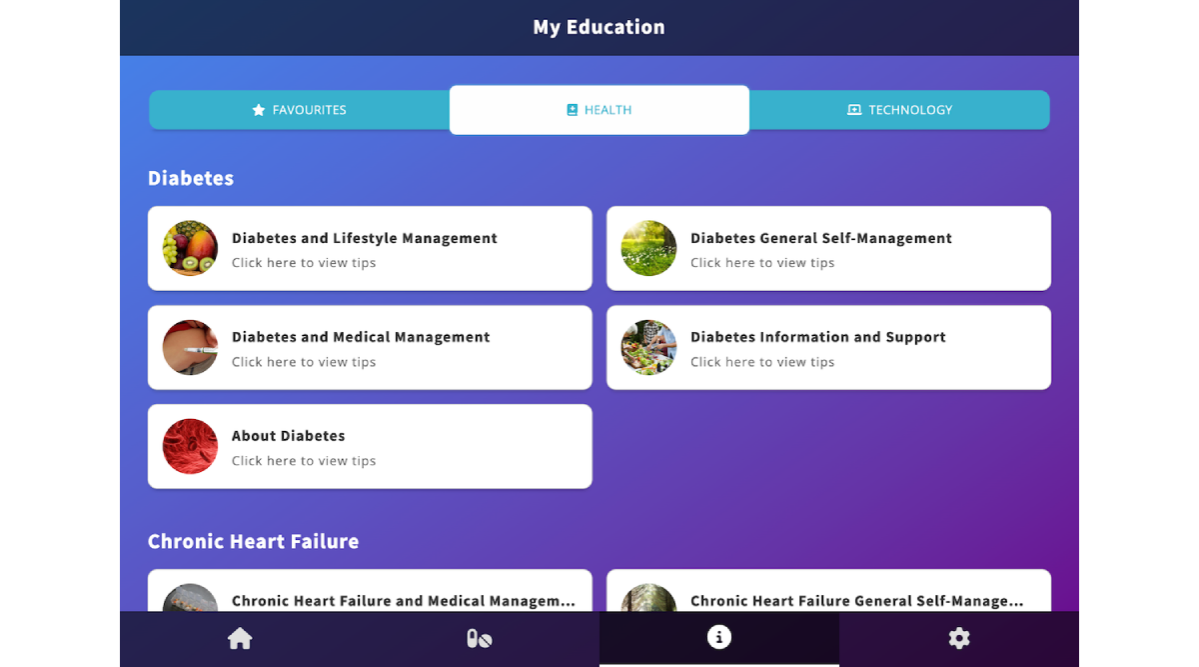
The education library showing content relevant to the user’s conditions and training content on using the technology (devices and CareApp).

Accessed via an interactive device (eg, a tablet or smartphone), this app displays users’ health and well-being data as derived from the different measuring devices. To support people with multimorbidity self-managing their health and well-being, the CareApp provides feedback (eg, feedback on health status including exacerbations), tools (eg, medication management tools and goal-setting features), and personalized educational content (eg, written and visual content sourced from relevant, reliable, and trusted organizations localized by region) that is contextualized to the person’s current health and well-being status. It also provides participants with the option to self-report through a series of questions about their health and well-being (eg, for those parameters not easily measured by a sensing device, such as breathlessness and mood). An overview of features is provided in Table 1 and Figures 1-5. The primary differences in the CareApp for those in trial arm 2 are that exacerbations are not highlighted, and educational content is not contextualized to the current status, therefore representing a more standard application that might be downloaded from an app store. People with multimorbidity will be the main users of the ProACT CareApp. However, customized ProACT CareApp interfaces will also be available for care network members of participants in trial arm 1 to use on their own devices (eg, tablets, smartphones, or computers) to view the participant’s data.

**Table 1:** Summary of features available within the ProACT CareApp for users with multimorbidity.

Component	Feature summary
Dashboard	Today at a Glance (Figure 1): A quick glance overview of the user’s current symptom (eg, weight and blood pressure) and well-being (eg, physical activity and sleep) status today, including any alerts detected, any measures missing (based on the participant’s care plan of how often they want to measure different parameters), progress toward an activity goal, and medications taken so far today.
Dashboard	Self-report: A series of symptoms (eg, breathlessness and chest tightness) and well-being (eg, mood and anxiety) questions. The self-report card (Figure 1) indicates how many outstanding questions are to be answered. Users can also see the responses to these questions charted.
Dashboard	My Health, My Wellbeing, My Activity: These sections of the dashboard present a series of cards, indicating the user’s most recent health and well-being readings (covering sensor and self-report data) or whether a reading is missing.
My Health, My Wellbeing, My Activity readings	Tapping on any of the cards in the My Health, My Wellbeing, or My Activity sections opens a detailed view of the user’s readings (Figure 2). This view includes a chart with a calendar for navigating between weeks, a summary of weekly readings (eg, highest, lowest, and number of alerts), a detailed list showing each reading with its timestamp and whether it fell within the normal range or triggered an alert, and 1 or 2 pieces of educational content relevant to the user’s current status for the parameter being viewed.
Activity or weight goal	In addition to being able to view activity and weight data over time, users can set a goal in relation to both parameters and track their progress (Figure 3). Daily or weekly activity goals can be set, while weight goals have no time frame.
Medication	The medication section of the CareApp allows users to add their medication list (prescription) including dosage, frequency, and any instructions for taking, as well as see their daily schedule and track when a medication has been taken (Figure 4).
Education	A library of education is included in the CareApp, which contains 2 main sections—content relevant to the conditions the user is managing and training content to help the user with using the devices and CareApp (Figure 5). Users can also add content to a favorites section.
Settings	This section allows users to set up their care network, to personalize certain settings (eg, to choose kilograms or stones and pounds for weight readings), and to choose between a light and dark theme.

#### Admin Interface

Admin interface is a user management system to allow researchers or service organizations to personalize CareApps to individuals and manage, inspect, and analyze data.

#### Data Flow Through the ProACT Platform

The flow of data through ProACT starts and ends with people with multimorbidity and care network members. The following steps occur in the data transfer process:

- The person with multimorbidity wears or interacts with a measuring device.
- For connected devices, data are automatically transferred to the device provider’s systems and then to the ProACT platform (ie, the ProACT CareApp front-end application and the admin back-end systems). For full details on the ProACT platform, see Doyle et al [24].
- For nonconnected devices, the person with multimorbidity manually enters their readings using the ProACT CareApp.
- On receipt of new data, the ProACT platform will check all values against a person with multimorbidity’s alerting thresholds. Where a threshold breach is encountered for a participant in trial arm 1, an alert is raised for the attention of the triage team. Triage teams can access, track, and categorize alerts through a provided administrative interface.

## Methods

### Study Design

#### Overview

This study comprises an EIH trial design in each country. In accordance with EIH philosophy, the objectives are (1) to assess the potential effectiveness of an intervention or interventions and (2) to assess their implementation, as well as factors associated with their implementation [28,30]. By assessing effectiveness and implementation concurrently, we can quickly learn about for whom, in what circumstances, and how an intervention or interventions work, if at all. In essence, the benefits include the efficient generation of research-to-practice translational gains, the identification of effective implementation strategies, and the development of relevant and useful information for policymakers and decision-makers (eg, the identification of barriers and facilitators to implementation can inform the selection of more appropriate strategies). A type 1 hybrid design will be implemented, which primarily focuses on effectiveness while exploring implementation. This design is recommended when the body of evidence in support of an intervention’s effectiveness is limited, and thus prioritizing implementation, and related barriers, facilitators, and strategies are premature.

The EIH trial will comprise a p-RCT to measure effectiveness and a process evaluation to measure implementation. p-RCTs are used to assess the effectiveness of an intervention in real-world conditions [31,32]. While traditional randomized controlled trials are used to assess the efficacy of an intervention in ideal circumstances, p-RCTs are instead used to ensure that external validity is prioritized (ie, to ensure that the findings are generalizable to the real world), with internal validity still preserved as much as possible. Furthermore, while the conduction of traditional randomized controlled trials ensures that participants are often exposed to scenarios that are considerably different from their everyday lives, the conduction of p-RCTs ensures that participants are exposed to real-world scenarios, such as real-world approaches to recruitment (eg, for targeted health care support), follow-up enquiry and support, and flexibility and tailoring of delivery to support needs. Process evaluations are used to assess the implementation of an intervention in real-world conditions [33,34], in particular to assess the fidelity and quality of implementation of an intervention in real-world conditions, and to identify and understand causal mechanisms and contextual factors that are associated with varying outcomes [35]. The study design of the p-RCT and process evaluation are outlined separately below.

#### p-RCT Design

A p-RCT trial will take place in each trial site with each person with multimorbidity and their care network participating for 6 months. A 6-month period is sufficient to observe changes in key outcome metrics and to ensure that the novelty of a new technology is not a confounding variable. A 6-month period also ensures that a staggered approach to recruitment over the 32-month period can take place, thus mitigating against potential challenges for large-scale recruitment and implementation of the technology. A 3-arm design will be implemented with participants with multimorbidity. Textbox 1 indicates the differences between the arms.

A mixed methods approach will be adopted, with experimental and descriptive assessments used to compare participants in trial arms 1, 2, and 3. A more detailed description of methodologies used in the p-RCT is outlined below.

**Pragmatic randomized controlled trial arms.**

**Trial arm 1**

- Tablet device and a suite of sensors
- Personalized ProACT CareApp
- Inclusion of the care network
- Clinical triage service support
- Technical helpdesk support

**Trial arm 2**

- Tablet device and a suite of sensors
- Standard ProACT CareApp with no personalization
- Technical helpdesk support

**Trial arm 3**

- Usual care (no technology)

#### Process Evaluation Design

##### Overview

The process evaluation to be implemented within SEURO will adopt a mixed methods approach informed by three main theoretical frameworks: (1) the Consolidated Framework for Implementation Research (CFIR) [36]; (2) a digital health technology model of transferability, ProTransfer, which was developed during the ProACT project [37]; and (3) the Reach, Effectiveness, Adoption, Implementation, and Maintenance (RE-AIM) framework [38]. The domains identified in these frameworks will be used to guide the development of the process evaluation. Below, we provide a brief description of each theoretical framework.

##### The Consolidated Framework for Implementation Research

The CFIR identifies five major domains of interest: (1) the outer setting, which includes the social, political, and economic context that the organization in which implementation is occurring exists; (2) the inner setting, which includes the features and characteristics of the organization such as leadership and relative priority; (3) the characteristics of individuals, which includes staffs’ knowledge and attitudes about the intervention, and their role and identification within the wider organization; (4) the characteristics of the intervention itself; and (5) implementation processes, which include the ways that the intervention will be delivered in a given context (including fidelity to the implementation strategy).

##### The ProTransfer Model

Informed by the CFIR, the ProTransfer model addresses factors influencing organizations’ transferability and implementation readiness for digital health technologies across four domains: (1) solution-specific factors, including adaptability (ie, the degree to which a solution can be tailored or refined to meet local needs), usability and design, trialability, and evidence of potential benefits and costs; (2) organization-specific factors, encompassing culture (ie, norms, values, and assumptions shaping acceptance and adoption), learning climate, leadership engagement, openness to change, resource availability, and compatibility with existing practices; (3) process-specific factors, such as engagement mechanisms (ie, attracting and involving key individuals through social marketing, education, role modeling, and training), formal leadership structures, participatory execution and evaluation, opinion leader engagement, and planning; and (4) individual-specific factors, including the self-efficacy of individuals involved.

##### The RE-AIM Framework

The RE-AIM framework identifies implementation outcomes across three domains: (1) reach—the proportion of eligible participants who open, use, and complete an intervention; (2) uptake—the proportion of eligible participants who opt in; and (3) acceptability or appropriateness—the perceived fit of the intervention.

**Figure 6:**
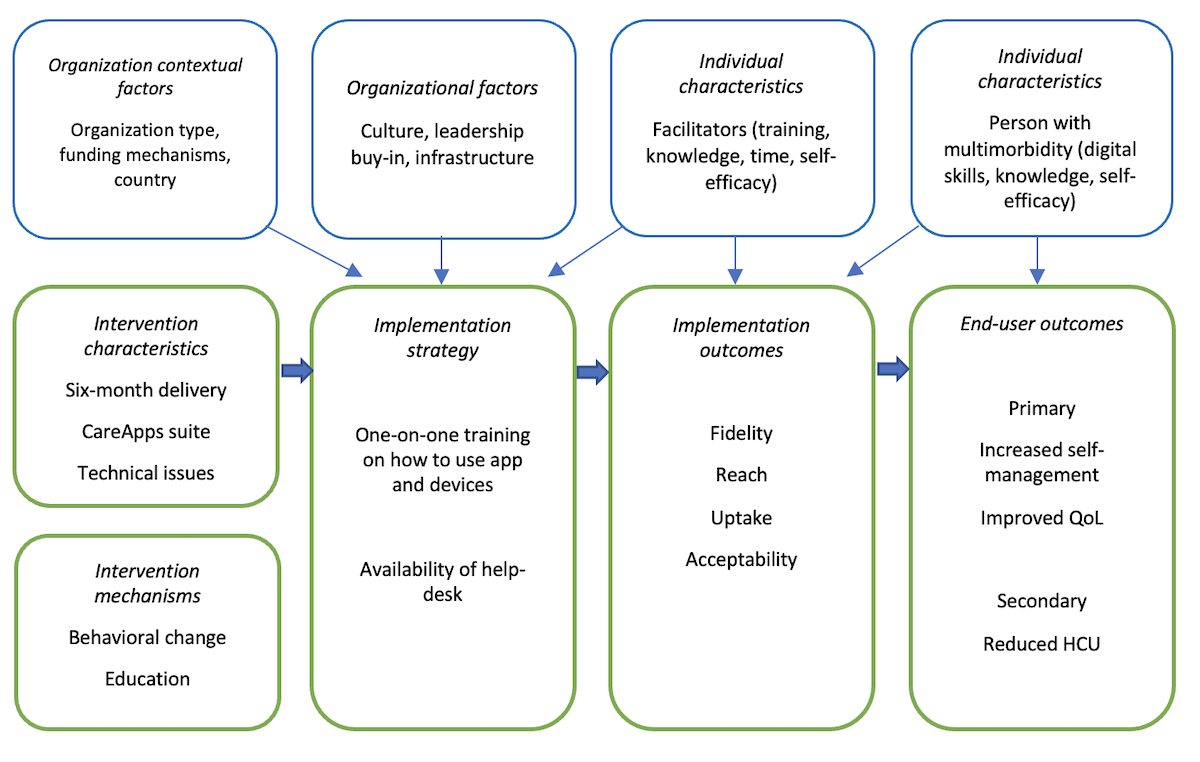
Process evaluation logic model. HCU: health care use; QoL: quality of life.

As outlined in the Medical Research Council guide to developing process evaluations [34], a logic model (Figure 6) was developed for SEURO based on prior ProACT Horizon 2020 research. It captures key factors influencing implementation and clinical effectiveness, integrating elements of CFIR, ProTransfer, and RE-AIM. Factors in blue represent those varying between organizations, triage staff, and people with multimorbidity and their care networks, while those in green represent consistent implementation characteristics across stakeholders.

The process evaluation at each trial site will explore (1) how ProACT is implemented, delivered, and used at the partner organization, clinical triage service, and participant levels (person with multimorbidity and care network participants); (2) systematic differences and variations in implementation, delivery, and use at these levels; (3) the reasons for these systematic differences and variations (ie, facilitators and barriers) at these levels; and (4) the relationship between implementation, delivery, and use and potential effectiveness in relation to perceived QoL, HCU, and user-observed self-management experiences. A more detailed description of the methodologies used in the process evaluation is outlined below.

### Sample

Three main participant groups will be recruited for the EIH trial in each of Ireland, Belgium, and Sweden: older people with multimorbidity, care network members, and partner organization management and staff involved in overseeing and implementing ProACT delivery. A maximum of 1920 participants (ie, people with multimorbidity and care network members) will be recruited across the 3 trial sites. This sample will consist of a maximum of 720 participants with multimorbidity (240 at each site) and a maximum of 1200 key individuals within the trial arm 1 participants’ care networks (400 at each site). Up to 15 partner organization managers or staff (5 at each site) will also be recruited.

### Participant Inclusion and Exclusion Criteria

Participants with multimorbidity must be aged 65 years or older, possess sufficient cognitive capacity to provide written informed consent, and have at least 2 of the following conditions: diabetes, chronic respiratory disease (eg, chronic obstructive pulmonary disease and chronic asthma), chronic heart failure, chronic heart disease (eg, coronary artery disease and cardiovascular disease, including hypertension, atherosclerosis, angina, or arrhythmia).

For care network participants to be included, the person with multimorbidity must provide consent for their participation. Care network participants must be aged 18-90 years; be providing support to a consented participant with multimorbidity in trial arm 1 (if they are an informal caregiver, they must be providing support to a participant with multimorbidity for at least 6 months; and if they are a formal caregiver, formal care quality assistant, or health care professional, they must be formally recognized as such and possess at least 2 years of experience of professionally working in a relevant health or primary care setting with people with multimorbidity with the same health conditions as those involved in this trial); have access to a tablet, smartphone, or computer with an internet connection; possess the skills to use an internet-based app or website; and possess sufficient cognitive capacity to provide written informed consent.

Partner organization management and staff must be aged 18 years or older; be overseeing and implementing the delivery of ProACT to their clients with multimorbidity; have access to a tablet, smartphone, or computer with an internet connection; and possess the skills to use an internet-based app or website.

### Recruitment Procedures

Study recruitment will take place from various sources across the 3 trial site countries. In Ireland, people with multimorbidity will be recruited from multiple sources, including local health service executive clinics, general practitioner (GP) clinics, home care organizations, private hospitals as well as through social media, radio and local newspaper advertising, and relevant older persons and chronic disease networks (eg, diabetes and chronic obstructive pulmonary disease support groups). People with multimorbidity in Belgium will primarily be recruited through a clinical partner, Z-Plus. Other recruitment sources will include GPs, hospitals, social media and local advertising, a third-party recruitment company, as well as older persons and chronic disease networks. Study recruitment in Sweden will take place in the municipality of Umeå in Region Västerbotten via multiple sources including local cardiology clinics and associated clinical connections, relevant older persons and chronic disease networks (eg, the Association for Disability and Chronic Disease and the older adult service “Senior Square”), and advertising (eg, distributed information leaflets and flyers). Each potential participant will take part in an initial phone call, where they will receive information about the study and complete a screening process to ensure that they meet the eligibility criteria. If they wish to participate and prove eligible to do so, the research team will then send an information pack to them by email or post, containing an invitation letter, a participant information leaflet (PIL), and a written informed consent form. The PIL will also include the research team’s contact details if the potential participants have any questions or concerns. A research team member will phone the potential participants 1 to 2 weeks (ie, 7 to 10 days) after sending the information pack to discuss their participation, provide additional information, and answer any questions. Those who express a willingness to participate will be asked to provide written informed consent, either using an online version or the hard copy version.

Across all 3 trial sites, participants with multimorbidity in trial arm 1 will be asked to identify up to 5 individuals within their care networks to take part in the study. They will be asked to provide these individuals’ contact details and to provide permission for the research team to contact these individuals. Each potential care network participant will be sent an invitation letter, PIL, and a written informed consent form, either by post or email. The study information materials will contain the research team’s contact details if the potential participants have any questions or concerns. A research team member will also contact them 1 to 2 weeks after sending these materials to discuss their participation, provide any additional information, and answer questions. Those who express a willingness to participate will be asked to provide written informed consent.

Managers or contact points in organizations that agree to take part in the study will be asked to identify members of their staff (including management themselves and clinical triage staff) to participate in the process evaluation (which will consist of completing 1 to 2 questionnaires and 1 interview). PILs and consent forms will be provided to process evaluation participants.

### p-RCT Methodology

#### Overview

A sample of 720 community-dwelling persons with multimorbidity across Ireland, Belgium, and Sweden (n=240 per site) will be recruited to take part. Participants will be randomly assigned to 1 of 3 equal-sized trial arms: overall, 240 will be assigned to each arm (representing 80 assigned to each arm in Ireland, Belgium, and Sweden, respectively). With the permission of participants with multimorbidity in trial arm 1, up to 5 individuals in their care network will also be invited to participate; however, participants in trial arm 1 can still participate without the inclusion of any care network representative.

#### Person With Multimorbidity Participants

All participants will complete baseline questionnaires capturing their demographic, QoL, health, psychological and psychosocial, HCU, and multimorbidity self-management information (Table S1 in Multimedia Appendix 2) [39-48]. To reduce participant burden, questionnaires can be completed over 10 days. Data will be collected and managed using REDCap (Research Electronic Data Capture) software [49,50], hosted at Vrije Universiteit Brussel. Data collection mode will depend on participant preference. Those comfortable with technology will receive an email link; others will receive paper questionnaires, returned by post or collected during home visits for technology deployment (trial arms 1 or 2). Researchers may assist participants during these visits. For trial arm 3 participants, assistance will be provided via phone or online call, with researchers recording responses. When calls are not possible, a home visit will be arranged. Up to 60 participants in each of trial arms 1 and 2 (20 per country in Ireland, Belgium, and Sweden) will also take part in qualitative interviews to capture baseline information on current self-management strategies and trial expectations. Interview themes are detailed in Table S2 in Multimedia Appendix 2, while the T1 interview protocol is outlined in Multimedia Appendix 3. Interviews will be audio-recorded, transcribed, annotated, and analyzed.

All participants will complete QoL and HCU questionnaires at trial start (T1) and end (T2). As HCU is a key outcome, participants will report monthly to improve recall with data captured through REDCap either online or via researcher-assisted calls.

At trial completion, participants will again complete questionnaires (Table S1 in Multimedia Appendix 2), within a 10-day period, but if extenuating circumstances exist (eg, if participants are on holiday), within a 4-week period. Semistructured interviews with up to 60 participants in each of trial arms 1 and 2 will explore experiences using ProACT, self-management strategies, symptoms, and care network roles (themes in Table S2 in Multimedia Appendix 2, full interview protocol in Multimedia Appendix 4).

System use data (eg, technology engagement metrics and health data from sensors) and triage service use (eg, call frequency) will be continuously collected through the ProACT platform throughout the 6-month trial (Table S2 in Multimedia Appendix 2).

The primary outcomes for participants with multimorbidity include QoL and HCU.

The secondary outcomes include self-management (ie, assessed via a mixed methods approach; eg, this includes an examination of participants’ perceived self-management using the custom-designed questionnaire and interviews with participants to explore their use of ProACT), health status (eg, an exploratory examination of the impact of ProACT on the hypothesized stabilization or reduction of participants’ symptoms using the system data), quality of health care support, and other issues of relevance include multimorbidity self-management knowledge, attitudes (eg, confidence in ability to self-manage), and skill; health literacy, digital literacy, and associated attitudes (eg, attitudes toward technology).

Other outcomes for participants with multimorbidity include: the usability, accessibility, and acceptability of, and engagement, satisfaction, and overall experiences with, ProACT.

#### Care Network Participants

Care network participants will be asked to complete questionnaires in the same manner as participants with multimorbidity (ie, online or over the phone, with data captured through REDCap [49,50]). At the beginning of their trial periods, all care network participants will be asked to complete a questionnaire that captures their demographic information; informal caregivers will also be asked to complete the Zarit Burden Interview [51]. At the conclusion of their trial periods, all care network participants will again be asked to complete 2 questions that capture their opinion on how the use of ProACT influenced their care and any burden they may have experienced while using ProACT (ie, measured via the User Burden Scale) [47]. Informal caregivers will also be asked to complete the Zarit Burden Interview [51]; while any formal caregivers, formal care quality assistants (ie, managers of formal caregivers), or health care professionals taking part in the process evaluation may have an additional questionnaire to complete (ie, the ProTransfer questionnaire; see Process Evaluation Methodology section). All questionnaires can be completed online or over the phone, depending upon the participants’ preferences. The full list of key assessment domains and measures is available to view in Table S3 in Multimedia Appendix 2.

At the conclusion of their trial periods, the research teams will also conduct semistructured interviews or focus groups with up to 60 participants in each care network group (ie, with up to 20 participants in each care network group in each country) to collect data on their satisfaction with and attitudes toward the technology, etc. These will likely take place over the phone or online due to trial resource limitations and the anticipated wide geographic spread of care network participants within each country, particularly of informal caregivers. For health care professionals and formal caregivers or formal care quality assistants, these will take place either by phone, online, or at their place of work, depending upon what is most convenient for them. The interviews or focus groups will be audio-recorded and transcribed, and the researchers will subsequently annotate and analyze the collected data.

Finally, care network participants’ system use statistics (eg, data that capture their use of the ProACT CareApp) will be collected continuously throughout their 6-month trial period.

The primary outcomes for informal caregivers include caregiver burden. Other outcomes for all care network participants include the usability, accessibility, and acceptability of, and engagement, satisfaction, and overall experiences with, ProACT.

### Process Evaluation Methodology

Participants with multimorbidity, care network participants, and the clinical triage services and partner organization staff will participate in the process evaluation. For participants with multimorbidity and care network participants, the process evaluation will not result in additional research activities beyond those described in Table 2 and Tables S1 and S2 in Multimedia Appendix 2. However, topics specific to the process evaluation will be included in the interview and focus group protocols for the p-RCT evaluation.

Four types of data will be collected: administrative data, self-report questionnaire data (eg, ProTransfer data), digital analytic data (ie, system use data), and interview data. Table 2 summarizes what data will be collected, from whom, and when. Some of the data listed here will already be collected during the p-RCT (eg, the digital analytics data). For the semistructured interviews or focus groups, up to 60 participants with multimorbidity in each of trial arms 1 and 2 (ie, up to 20 participants in each country), up to 60 individuals in each care network group (ie, up to 20 individuals in each care network group in each country), the clinical triage services, and up to 15 partner organization staff (ie, up to 5 staff in each country) will be asked to participate. Interviews will likely take place over the phone or by online video call. The interviews and focus groups will be audio recorded and transcribed, and the researchers will subsequently annotate and analyze the collected data. The administrative, ProTransfer (questionnaire), and digital analytics data will be captured using electronic means (ie, online).

The outcomes for participants with multimorbidity in trial arms 1 and 2, all care network participants, and the clinical triage nurses include the usability, accessibility, and acceptability of, and engagement, satisfaction, and overall experiences with ProACT; and the way end users (ie, participants with multimorbidity, care network participants, and the clinical triage nurses) use ProACT (eg, to view one’s readings, to set goals, and to consume educational material), how they differ in this regard (eg, to view readings only, to set goals only, or to consume educational material only), and the factors that influence these differences (eg, a participant with multimorbidity’s condition and age).

The outcomes for partner organization staff include the reach, uptake, and fidelity of implementation of ProACT; the usability, accessibility, and acceptability of ProACT; and engagement, satisfaction, and overall experiences with ProACT; and the way organizations implement and deliver ProACT, how they differ in this regard, and the factors underpinning these differences (eg, societal- and organizational-level contextual factors, such as leadership buy-in).

**Table 2:** Process evaluation data collection.

Participants	Administrative data	ProTransfer data	Digital analytics	Interviews or focus groups
Management	✓ (pre)	✓ (pre and post)		✓ (post)
Participants with multimorbidity and care network participants			✓ (ongoing)	✓ (post)
Clinical triage nurses			✓ (ongoing)	✓ (post)

### Trial Procedures

The ProACT technology, described earlier and in Table 1, will be deployed to trial arm 1 and 2 participants in their homes for 6 months. Participants will regularly use the measuring devices and ProACT CareApp to record and manage key health and well-being parameters. Depending on the device, data will be uploaded automatically to the CareApp via Wi-Fi or Bluetooth or entered manually via the CareApp. An Internet connection will be provided to participants who do not have an existing connection in their homes. The technology will be withdrawn at the end of the trial, which will be clearly communicated beforehand and outlined in the consent form and PILs.

Care network participants will be asked to use the CareApp to view data shared by the participants with multimorbidity and to engage with the provided education materials. More specifically, the CareApp interface for each care network participant will allow the user to view the health and well-being data and medication details of participants with multimorbidity. Informal caregivers and formal caregivers will also receive educational material that pertains to the conditions of the participants with multimorbidity that they care for. Care network participants will be able to use the ProACT CareApp at a time and with a frequency that is convenient to them. The aim is to explore their experiences of technology and its perceived usefulness in supporting people with multimorbidity.

For trial arm 1, a clinical triage service in each country will review participants’ vital signs and alerts at least 5 days a week (Monday to Friday). Triage nurses may adjust personalized thresholds and will contact participants if alerts are triggered, to discuss the alerts, and to determine if further action is required. For instance, the participants will be encouraged to bring their CareApp to their health care visits (eg, in situations where a health care professional has not consented to participate in the trials); where a health care professional has consented to participate, they will be able to view the relevant data in their CareApp, including detailed weekly data across all health and well-being parameters.

The clinical triage services will also support the development and personalization of data collection plans for participants in trial arm 1. Prior to the trial, the research and triage teams will create a ProACT data collection plan “care plan” (for data collection only rather than for clinical or treatment purposes) for participants in trial arm 1, tailored to their conditions (eg, how often they should measure their symptoms). As the trial progresses, the clinical triage services, in consultation with participants, will personalize their data collection plans. Participants can access their plans through their CareApp and share them with those in their care network.

### Sample Size Justification

We used a 3-group comparison (ie, trial arms 1, 2, and 3) as the main fixed effect for a repeated measures ANOVA model to determine the sample size of the p-RCT. For a desired power of 0.80, an error variance of 2, and a type I error rate of 0.05 with an attrition rate of 30%, we estimated that we need 76 participants per arm per trial site (rounded up to 80) to detect a standardized effect size (Cohen *d*) of 0.25. In the situation that recruitment targets have been met and more participant candidates can be included in the study, we will aim for a sample size of 100 per arm per trial site instead of 80 to obtain a power of 0.90. Sample size and power analysis were conducted using STATA (version 17.0; StataCorp LLC).

### Randomization

Upon study enrollment, the participants with multimorbidity at each site will be randomly assigned to 1 of 3 equal-sized trial arms (ie, 80-100 participants with multimorbidity will be assigned to each arm). Block randomization will be implemented to ensure balance in the sample size across groups; given that there are 3 arms, a block size of 9 will be implemented (ie, participants will be block randomized per every 9 individuals). The block randomization will be set up in REDCap [49,50].

Following CONSORT (Consolidated Standards of Reporting Trials) guidelines for p-RCTs [52], this trial will be unblinded. Blinding the researchers to participants’ trial arms will not be possible due to the clear differences in the technology components across groups (eg, no component will be assigned to participants in trial arm 3). Additionally, it will not be possible to blind participants to the allocation of study or technology components.

### Data Analysis Plan

For the p-RCT primary outcomes, three types of analysis will be conducted: (1) What is the influence of ProACT on perceived QoL? (2) What is the influence of ProACT on HCU? (3) What trial arm is the most cost-effective?

To determine if significant differences exist in (1) perceived QoL between the arms and (2) HCU between the arms, a linear mixed model will be used. In this model, a 2-level structure will be applied; repeated measures of QoL or HCU will comprise the first level, and participants will comprise the second level in the hierarchy. The outcomes will be assessed longitudinally using fixed effects of time, treatment arm and time interaction, and other baseline covariates such as age and gender.

ProACT’s cost-effectiveness will be determined with the use of 2 economic evaluation techniques, cost-effectiveness standard and cost-utility analysis, applying the societal perspective. Societal perspective means, in general terms, the effectiveness evaluation will consider all the relevant contributors related to the participant. The 2 methods will allow the determination of the monetary value and the benefits of the intervention in health terms. For the monetary value, the HCU data will form the basis of the analysis.

Due to the questionnaire construction variation (countable and binary questions) and the time points of data collection during the trial, the analysis will make use of 3 statistical models: Poisson (countable data), logistic (binary response data), and linear regression (continuous data). The analysis will allow the observation of how ProACT affects the HCU, per arm at each trial site, and what the costs involved in this effect are for the monetary value definition. To define a 95% CI for this cost-effectiveness estimate per arm and per trial site, a sensitivity analysis will be performed, for instance, using a bootstrapping methodology.

Indirect costs of the care network (informal care) will be calculated using data collected from the participants with multimorbidity and their informal caregivers. First, participants with multimorbidity will provide data on time spent on care by their informal caregivers. With the use of the opportunity costs method, this time will be converted into monetary value. Next, data collected from the informal caregivers, via the Zarit Burden Interview, will facilitate an assessment of the health gains of the time spent in care, considering the ProACT intervention. Indirect costs will be considered as an aggregated cost in this study.

The cost-utility analysis technique will underpin the quality-adjusted life years calculation. The quality-adjusted life years is a general measure used in economic evaluation that permits the valuation and comparison of the health gains of an intervention. For this calculation, the health-related QoL measurement instrument (EQ-E5-5L) will capture the self-perceived health status of the participant in a 5-digit code. Next, the code will be converted into a utility value (or utility weight), based on the country-specific value set. The result consists of the multiplication of the utility value with the period lived with the self-perceived condition.

For the secondary outcomes, such as the impact of ProACT on self-management, we may use structural equation modeling to determine the relevance and importance of different variables measured during the trial. Hitherto, there is no consensus on how to measure improved self-management. For this study, we may consider the concept of self-management as a composite measurement of different variables proven to be associated with the ability to self-manage chronic illnesses. Variables that are associated with improved self-management include, for instance, self-efficacy, health literacy, and disease knowledge, and therefore may be treated as latent variables. Using a structural equation model, such as confirmatory factor analysis, we will be able to identify the existence of a relationship between these variables and an overarching concept of self-management.

Outcomes from this exploratory analysis will help to determine whether features in ProACT could be tested in future clinical trials. Outcomes from the process evaluation and their inclusion, where possible, in the aforementioned exploratory analysis may also help to determine the relationship between implementation and effectiveness, and for whom, in what circumstances, and how ProACT influences the primary and secondary p-RCT outcomes. Descriptive summary statistics will be collated on the primary and secondary outcome measures, as well as on the variables included in the exploratory analysis.

The process evaluation will draw upon a range of quantitative and qualitative data to explore the implementation of ProACT and the factors that impact upon it. Descriptive summary statistics will be collated and examined where applicable (eg, demographic and system use data for participants with multimorbidity and care network participants, partner organizations’ administrative and ProTransfer data, and participant recruitment and withdrawal data).

Interview and focus group data will also be attained to explore implementation and factors associated with it, including, for example, the perceived usability, accessibility, and acceptability of, and engagement, satisfaction, and overall experiences with, ProACT. The interviews and focus groups will be audio recorded, transcribed, and imported into NVivo (QSR International; in Ireland and Sweden) and MAXQDA (VERBI GmbH; in Belgium) for analysis. The researchers will also take brief notes during the interviews and focus groups to aid data interpretation and to identify potential themes. The interdisciplinary research team, with expertise spanning social, health, and sports psychology, human-computer interaction, and health care innovation, will actively engage in reflexive practice throughout the analysis. This collaborative approach will ensure participant voices are central while critically accounting for researchers’ potential biases shaped by their backgrounds.

Collaborative qualitative analysis will be used to thoroughly examine interview data, leveraging multiple researchers’ perspectives to capture nuances, manage large datasets, and reduce individual biases [53-55]. A hybrid thematic analysis approach will be used, combining inductive and deductive techniques based on Fereday and Muir-Cochrane’s framework [56]. The research questions, an initial literature review, and a preliminary examination of interview data shaped the deductive approach’s creation of a preliminary codebook. Simultaneously, the inductive approach will be used to identify new themes during coding, enriching the analysis. The transcripts will be coded using thematic categorizations, including within and between groups where applicable (eg, for trial arms 1 and 2 participants and for staff in different partner organizations).

The collaborative qualitative analysis framework will be applied with six sequential stages, commencing after the collection and transcription of the data: (1) preliminary organization and planning, (2) open and axial coding, (3) development of a preliminary codebook, (4) pilot testing the codebook, (5) final coding, and (6) reviewing the codebook and finalizing the themes. Consensus meetings will be conducted within and across trial sites to prepare the codebook and collaboratively identify and refine the themes. This approach ensures that diverse perspectives are considered, enhancing the validity and richness of the analysis throughout the research process.

### Ethical Considerations

The trial protocol has received ethics approval from 5 research ethics committees in Ireland, including the School of Health and Science at Dundalk Institute of Technology, Trinity College Dublin Faculty of Health Sciences (reference 220504), Health Service Executive Northeast (reference REC/22/026), Caredoc, and Blackrock Clinic. In Belgium, the protocol has been approved by the medical ethics committee of the University Hospital Brussels (approval EC-2022-246), while in Sweden, it has been approved by the Swedish Ethical Review Authority (approval 2023-07594-01). Potential participants will receive a PIL. All recruited participants will be required to give informed consent. Efforts will be undertaken to ensure that participants fully understand the study aims, procedures, and their rights, including the voluntary nature of participation and their right to withdraw at any time without consequence. Participants will not receive compensation for taking part. Participants will be informed about the collection and use of their personal data as per General Data Protection Regulation requirements. Comprehensive measures will be implemented to maintain participant privacy and data security, including data minimization, secure storage, controlled access, and deidentification of research datasets. This will include purposes for processing personal data, retention periods, and who data will be shared with. Data will only be shared with trial partners. Qualitative interview or focus group data and quantitative questionnaire data will be coded to participant IDs, with all qualitative transcripts reviewed and any identifying information removed. Electronic sensor data captured through the ProACT platform will be both coded and identifiable. The data that flow from the measuring devices to the device provider systems and admin platform will be coded. These data will be made identifiable within the admin platform to trial researchers as well as clinical triage nurses. It will also be identifiable on the CareApp interfaces of health care professionals and formal caregivers, if a participant agrees to this. For instance, a GP may have several patients using the ProACT platform, who will be listed in the GP’s ProACT CareApp. The GP will need to identify each patient by name. The health care professionals’ or formal caregivers’ access to their CareApp will be password-protected. The ProACT platform has been developed in compliance with General Data Protection Regulation requirements, and access will be managed and available to only those members of the teams who require access to conduct the trial and research. As the ProACT platform is a low-risk, noninvasive digital health intervention study, no formal ancillary or posttrial clinical care will be required. Participants will continue to receive routine care from their usual health care providers throughout and following their involvement in the study. Participants in the intervention arms will be informed that access to the ProACT platform and triage support (where applicable) will be time-limited to the duration of the study. Should any participant experience distress or identify health concerns during the trial, they will be advised to consult their GP or relevant care team, with trial staff trained to signpost participants to appropriate services when needed. Any significant amendments to the SEURO study protocol (eg, changes to study design, eligibility criteria, outcome measures, data collection procedures, or ethical considerations) will be formally documented, including a justification for the change and the potential implications for participants or study conduct. Amendments will be submitted to the relevant research ethics committees for review and approval prior to implementation, in accordance with national and institutional regulations. All approved amendments will be recorded in the trial registry (eg, ClinicalTrials.gov or EU Clinical Trials Register, if applicable) and communicated to all study sites, collaborators, and funders. Participants will be informed of any changes that may impact their involvement in the study, and reconsent will be sought where appropriate.

## Results

**Figure 7:**
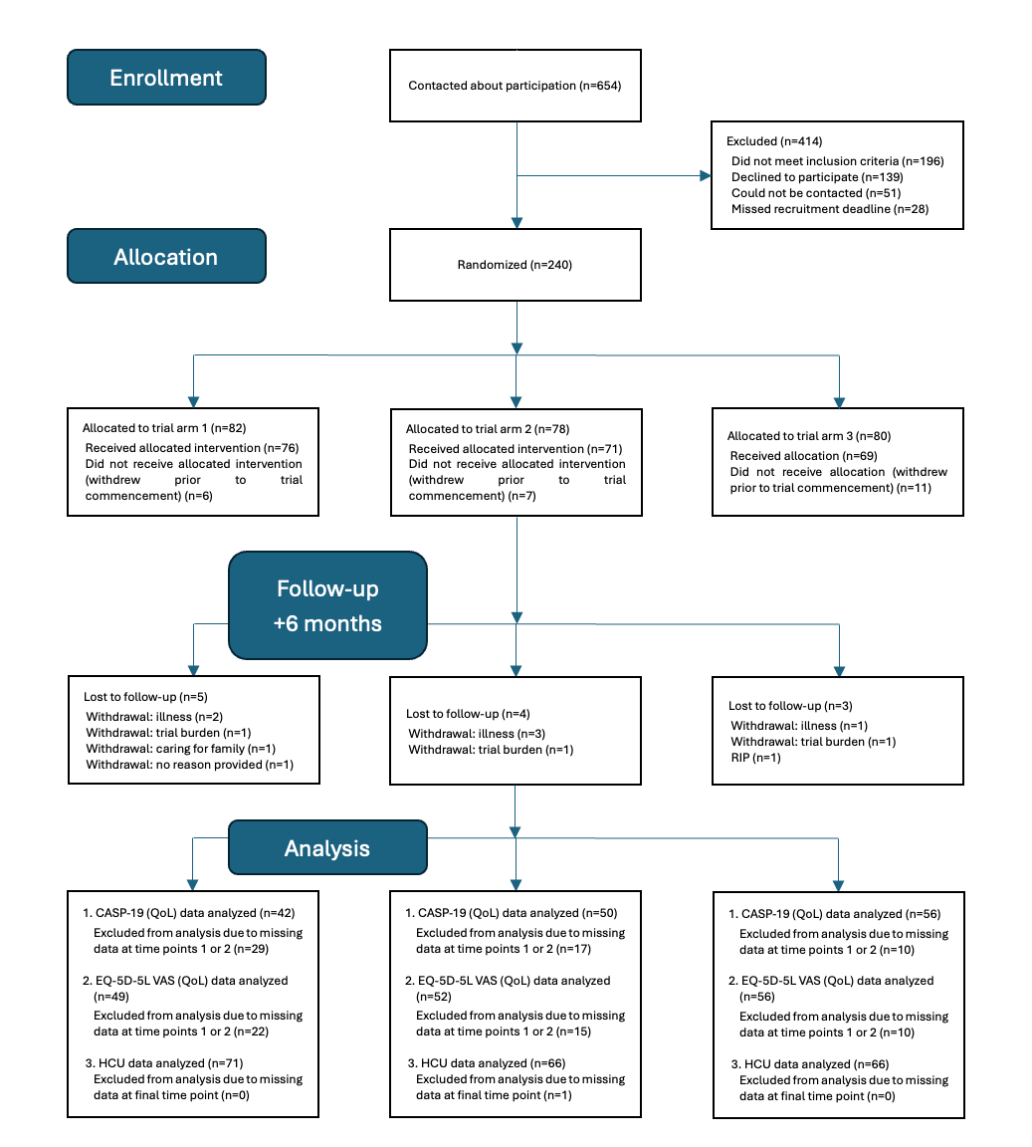
CONSORT diagram of Ireland participants with multimorbidity. CASP-19: Control, Autonomy, Self-realisation, Pleasure; HCU: health care use; QoL: quality of life; RIP: rest in peace.

**Figure 8:**
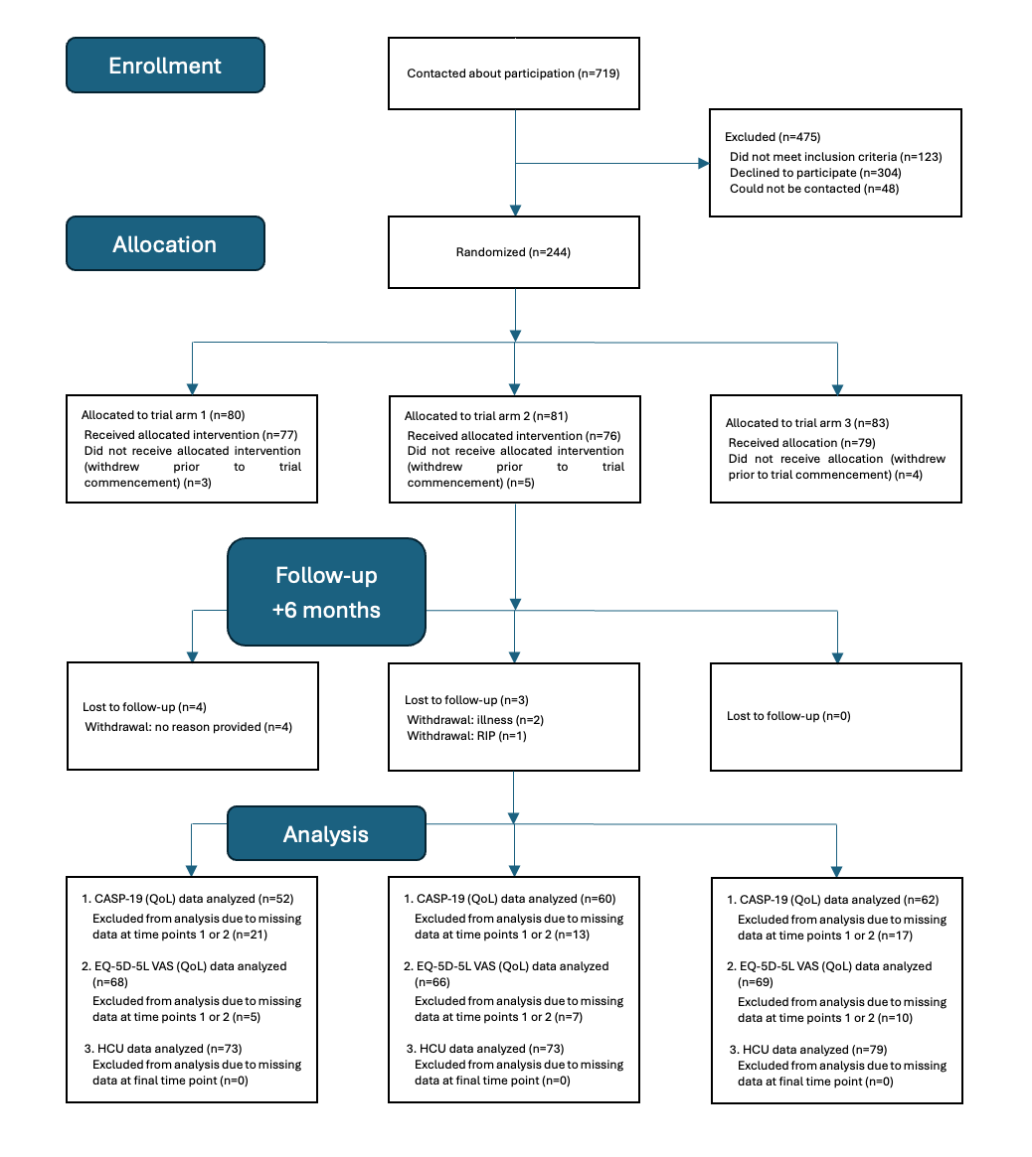
CONSORT diagram of Belgium participants with multimorbidity. CASP-19: Control, Autonomy, Self-realisation, Pleasure; HCU: health care use; QoL: quality of life; RIP: rest in peace.

Enrollment in the trial began in September 2022. To date, the trial has been completed in Ireland and Belgium. CONSORT diagrams are provided in Figures 7 and 8, depicting the recruitment and flow of participants with multimorbidity through the trials in Ireland and Belgium, respectively. Recruitment in Sweden completed in October 2025, with final participants finishing the trial by March 2026. Trial outcomes will be submitted for publication in 2026.

## Discussion

### Principal Findings

This paper presents the protocol for the SEURO EIH trial, which will investigate the effectiveness and implementation of the ProACT platform. To the best of our knowledge, this is the first trial of its kind to evaluate the effectiveness and implementation of a digital health platform with older adults with multimorbidity. Recent studies have underscored the lack of comprehensive reviews on the implementation and evaluation of digital health platforms, noting that existing reviews adopt a narrow focus, often limited to single chronic disease management or specific elements of implementation [57,58]. The ProACT proof-of-concept trial [24,26,27] demonstrated that the digital health platform ProACT, designed with and for older people with multimorbidity and their care networks, facilitated perceived improvements in their health and well-being, self-management, and receipt of support. Building on the proof-of-concept trial, the SEURO EIH trial will provide a more definitive and in-depth assessment of the updated and refined ProACT platform as a digital solution to support multimorbidity management in older adults. The SEURO trial also builds on prior work by incorporating nurse triage support and assessing the impact of this support as a means of augmenting engagement and health and well-being outcomes. Finally, this trial will also facilitate the achievement of wider SEURO project objectives: namely, to evaluate the key factors necessary to prepare any European Union region to successfully implement and scale innovative, people-centric, digital integrated health and social care solutions for multiple disease management.

### Strengths

The SEURO EIH trial will incorporate a p-RCT and process evaluation to assess the effectiveness and implementation of the ProACT platform, respectively, in real-world conditions. This concurrent assessment of effectiveness and implementation will help us to understand if the platform (and related services) works as intended, and for whom, in what circumstances, and how (eg, we may learn about the facilitators of and barriers to use by certain persons with multimorbidity and identify strategies that incorporate and overcome these facilitators and barriers, respectively). Comparing the effectiveness and implementation of (1) the personalized ProACT CareApp, care network support, and clinical triage service support; (2) the standardized ProACT CareApp, absence of care network, and clinical triage service support; and (3) standard care could also provide valuable information about for whom, in what circumstances, and how a particular platform might be most suitable for certain persons with multimorbidity or certain health care organizations. The adoption of rigorous research methodology—for example, the recruitment of a diverse, large sample of older adults with multimorbidity across 3 European Union countries, and the collection of quantitative and qualitative data from various stakeholders (ie, persons with multimorbidity and their caregivers, health care organization management and staff, and clinical triage nurses)—will also help to assess the platform, to further build upon the ProACT proof-of-concept trial findings, and to advance this field of research more generally. As noted, few studies have tested the effectiveness and implementation of digital solutions for multimorbidity management, instead choosing to focus on design recommendations only. The SEURO EIH trial will address this gap.

An assessment of the ProACT platform will represent an efficient use of research resources, given its known likely benefits. Previous iterations of the platform underwent an extensive design and testing process during the ProACT proof-of-concept trial, which demonstrated positive outcomes. The platform was specifically designed to address the complexity of multimorbidity, incorporating behavioral change and human-computer interaction theory. It proved accessible and engaging for older adults with varying physical and mental capacities and was perceived to support improvements in their health and well-being. The current iteration has been further refined based on feedback gathered during and after the proof-of-concept trial. As such, its use and implementation hold the potential to generate positive outcomes—such as improvements in QoL and HCU among people with multimorbidity, reduced caring burden for informal caregivers, and increased satisfaction among health care organizations. Overall, the findings of this trial may also provide important information on next steps, such as how to further refine and modernize the ProACT platform, what strategies facilitate its fidelity of use and implementation, and the potential value of conducting larger clinical trials. Via the implementation of a systematic program of analysis of the collected data, with the findings published in peer-reviewed journals and presented at conferences, we will generate insights and advance the knowledge base of this emerging domain (ie, digital-health supported management of multimorbidity).

The generation of evidence-based support for the routine use of the ProACT platform in applied settings would represent considerable impact. With health care services under increasing strain and traditionally designed to support those with single morbidities, and with the emergence of threats such as COVID-19, it is more important than ever to develop actionable insights and resources that can empower persons with multimorbidity to self-manage their complex care needs at home, with the support of their caregivers. However, while interest in the use of digital solutions to support multimorbidity management is growing, its uptake is still limited, an issue that is perhaps partly attributable to the previously described research limitations. Bridging the research-practice divide via the ProACT platform could somewhat resolve this issue.

We anticipate that participants receiving triage support will demonstrate greater and more sustained engagement with the platform as well as improved symptom awareness and confidence in self-management. These findings could support the case for embedding light-touch clinical oversight in digital health services, especially for populations managing complex health needs. If successful, the approach could be scaled through integration with existing community nursing structures or primary care pathways, reducing the burden on acute services while preserving person-centered support. Additionally, insights from the Irish, Belgian, and Swedish contexts will inform strategies for adapting such services to different health system infrastructures.

### Limitations

There are some study limitations that should be considered. First, HCU data will be self-reported by participants rather than collected through health care organization records. While self-reporting offers valuable insights into participant perceptions and experiences, it introduces potential recall bias, particularly for older adults managing multiple conditions, who may struggle to accurately recall the frequency or nature of health care contacts. Additionally, self-reported data may be underreported or overreported depending on individuals’ health literacy, understanding of health care services, or social desirability bias. Some outcome measures, including digital engagement and symptom monitoring, will also rely heavily on quantitative use logs, which, while useful, may not always reflect the quality or intentionality of engagement. These limitations should be considered when interpreting the findings and drawing conclusions about the broader scalability of the ProACT platform. Finally, due to the nature of the study design, blinding of researchers during implementation will not be feasible, which may introduce potential bias. To mitigate this risk, researchers will adhere to predefined intervention protocols to minimize any influence on study outcomes.

### Conclusions

The SEURO EIH trial protocol provides a detailed account of the objectives, scientific procedures, and practicalities of preparing for and managing the trial. Given that it will take place in multiple European Union countries, this protocol ensures that the research teams at all sites will possess a shared understanding of what the trial entails and can conduct the trial in a coordinated and intended way. Any deviations from the protocol during the trial will also be transparent and open to scrutiny.

## Appendix

Multimedia Appendix 1 SPIRIT checklist.

Multimedia Appendix 2 Participant with multimorbidity and care network assessment domains.

Multimedia Appendix 3 T1 interview guide for participants with multimorbidity.

Multimedia Appendix 4 T2 interview guide for participants with multimorbidity.

Multimedia Appendix 5 Peer review report from Horizon 2020 - Research and Innovation Framework Programme, European Commission (EU).

## Abbreviations

CFIR: Consolidated Framework for Implementation Research
CONSORT: Consolidated Standards of Reporting Trials
EIH: effectiveness-implementation hybrid
GP: general practitioner
HCU: health care use
PIL: participant information leaflet
p-RCT: pragmatic randomized controlled trial
QoL: quality of life
RE-AIM: Reach, Effectiveness, Adoption, Implementation, and Maintenance
REDCap: Research Electronic Data Capture
SEURO: Scaling European Citizen Driven Transferable and Transformative Digital Health
SPIRIT: Standard Protocol Items: Recommendations for Interventional Trials

## Data Availability

The datasets generated or analyzed during this study are not publicly available due to ethical and privacy considerations but are available from the corresponding author on reasonable request, if approval is obtained from the relevant ethics committee and data sharing complies with institutional and legal guidelines.
