# Supplementary material for: Scaling European Citizen Driven Transferable and Transformative Digital Health: Protocol for an Effectiveness-Implementation Hybrid Trial of a Digital Health Platform to Support Multimorbidity Self-Management
Source: JMIR Res Protoc. 2025 Nov 25;14:e74989. doi: 10.2196/74989 (PMC12690278; doi:10.2196/74989)
Supplement: Multimedia Appendix 1 [file resprot_v14i1e74989_app1.pdf]

SPIRIT 2013 Checklist

SPIRIT Item	Description	SEURO Protocol Compliance
1. Title	Descriptive title with trial acronym	Present on first page: ‘Scaling EUROpean citizen driven…’
2. Trial registration	Trial identifier and registry	ISRCTN34134007 provided in abstract
3. Protocol version	Date and version identifier	Version 2. Details and dates in trial registry. One update made to protocol on registry in July 2024.
4. Funding	Sources and types of financial support	Acknowledged: EU Horizon 2020, GA no. 945449
5. Roles/responsibilities	Names and affiliations of contributors	Author list and affiliations included
6. Background and rationale	Scientific background and rationale	Detailed in ‘Introduction’
7. Objectives	Specific objectives or hypotheses	Clearly listed in ‘Study Aim and Objectives’
8. Trial design	Description of trial design	Described as 3-arm p-RCT in ‘Methods’
9. Study setting	Description of study settings and sites	Provided under ‘Recruitment procedures’
10. Eligibility criteria	Inclusion and exclusion criteria	Separate criteria provided for each participant type in ‘Participant inclusion and exclusion criteria’
11. Interventions	Description of interventions	Detailed descriptions in ‘Trial Arms’ and ‘ProACT platform’
12. Outcomes	Primary and secondary outcomes	Listed in multiple tables (Tables 2–5)
13. Participant timeline	Schedule of enrolment, interventions, assessments	Described under ‘Trial procedures’
14. Sample size	Sample size determination and justification	See ‘Sample Size Justification’
15. Recruitment	Strategies for participant enrolment	Detailed by country in ‘Recruitment procedures’
16. Allocation sequence generation	Randomisation method	Block randomisation described in ‘Randomisation’
17. Allocation concealment mechanism	How allocation will be implemented	Via REDCap system, described in ‘Randomisation’
18. Blinding	Who is blinded and how	Not blinded – rationale and mitigation noted in

		‘Randomisation’
19. Data collection methods	Plans for assessment and collection of data	Comprehensive: questionnaires, system data, interviews, noted in ‘Methods’
20. Data management	Plans for data entry, coding, and storage	REDCap and ProACT platform used; GDPR compliance described
21. Statistical methods	Planned statistical methods	Linear models, cost-effectiveness analysis, SEM outlined. Addressed in ‘Data Analysis Pla,’
22. Research ethics approval	Plans for ethics approval	Ethics approved in 3 countries. Addressed in ‘Ethical Considerations’
23. Protocol amendments	Plans for communicating protocol changes	Addressed in ‘Ethical Considerations’
24. Consent or assent	Who will obtain consent and how	Described in detail under ‘Recruitment procedures’
25. Confidentiality	How personal information is protected	Covered extensively in ‘Ethical considerations’
26. Declaration of interests	Competing interests	Declared: ‘None’
27. Access to data	Who will have access to the final dataset	Addressed in data sharing statement at end of paper.
28. Ancillary and post-trial care	Provisions for post-trial care	Addressed in ‘Ethical Considerations’
29. Dissemination policy	Plans for trial results and authorship	Plans outlined in ‘Results’ and ‘Discussion’
30. Informed consent materials	Model consent forms and materials	Mentioned in ‘Recruitment’
31. Biological specimens	Plans for collection and use	Not applicable – no specimens collected
