# Supplementary material for: Scaling European Citizen Driven Transferable and Transformative Digital Health: Protocol for an Effectiveness-Implementation Hybrid Trial of a Digital Health Platform to Support Multimorbidity Self-Management
Source: JMIR Res Protoc. 2025 Nov 25;14:e74989. doi: 10.2196/74989 (PMC12690278; doi:10.2196/74989)
Supplement: Multimedia Appendix 2 [file resprot_v14i1e74989_app2.docx]

**Table S1**. Key assessment domains and measures captured in questionnaires for participants with multimorbidity.

| **Domain** | **Measure(s)** | **Arms** | **Time Point** | **Rationale** |
| --- | --- | --- | --- | --- |
| Demographics | - Information on gender, date of birth, ethnicity, marital status, educational level, living alone / with others, employment status, primary occupation | 1, 2, 3 | T1 | To perform inferential statistics and to include as covariates during analysis of primary and secondary outcomes |
| Comorbidity Index | - Type and number of comorbidities and perceived burden of comorbidities | 1, 2, 3 | T1, T2 | This data will provide insight into how self-management influenced the perceived burden of comorbidities. This data will be used during the cost-effectiveness analysis |
| Impact evaluation | - Custom questionnaire to assess participants’ opinions on the utility of the ProACT platform | 1, 2 | T2 | To capture participants’ perceptions of their experiences with ProACT (e.g., in relation to disease management, HCU increase/decrease, and/or care support independence). This data may be used to support exploitation strategies and business opportunities / new business models |
| Self-management:  Digital health literacy & knowledge | - HLS-EU-Q16 [40] - EHeals [41] | 1, 2, 3 | T1, T2 | When using ProACT, participants may learn about their diseases and improve their disease-specific management |
| Quality of life | - EQ-5D-5L [42] - CASP-19 [43] | 1, 2, 3 | T1, T2 | Quality of life measures are used to determine quality adjusted life years (QALYS). Quality of life is one of the primary outcomes of this study |
| Healthcare utilisation (HCU) | - Custom questionnaire on HCU over past month | 1, 2, 3 | Monthly | HCU information is required to determine the potential cost-effectiveness of the ProACT platform. Monthly data capture minimizes the recall bias and ensures more accurate cost calculations. HCU is one of the primary outcomes of this study |
| Self-management effectiveness:  Medication | - 5-item Medication Adherence Report Scale (MARS-5) [44] | 1, 2, 3 | T1, T2 | Improved adherence to medication is considered to be an aspect of self-management. This data will help to determine if self-management is improved when using the ProACT system |
| Self-management:  Self-efficacy, self-care & health literacy | - Self-Care Chronic Illness Inventory [45] | 1, 2, 3 | T1, T2 | These questionnaires capture participants’ self-efficacy, self-management and health literacy as they relate to dealing with comorbidities: all important attributes of multimorbidity self-management |
| Technology use and proficiency / digital literacy | - The Mobile Device Proficiency Scale [46] | 1, 2 | T1, T2 | Participants might improve their digital skills by using ProACT. Technical skills might also be important in understanding the relationship between the adoption and effectiveness of the self-management technology |
| Technology adoption and acceptance | - UTAUT-2 questionnaire [47] | 1, 2 | T2 | From an implementation science perspective, it is important to investigate how likely it is that participants will adopt the ProACT technology, besides exploring its effectiveness |
| Usability | - User Burden Scale [48] | 1, 2 | T2 | Data collected with the User Burden Scale can help us to determine if and what user experience issues prevent adoption of the technology |
| Health support system | - Custom questionnaire to assess the type and strength of participants’ health support system | 1, 2, 3 | T1, T2 | This data will provide insight into how the self-management of participants is affected by their social context. It can help to understand the social networks of participants and how it impacts their use of ProACT. |
| Expectations of Aging (To be measured in Belgium only) | - - The ERA-12 [49] | 1, 2, 3 | T1, T2 | This data will provide insight into participants’ expectations regarding aging |

**Table S2.** Key assessment domains and measures captured through system data and interviews for participants with multimorbidity (Trial Arms 1 & 2).

| **Domain** | **Measure(s)** | **Assessment Timepoint** |
| --- | --- | --- |
| ProACT engagement | - Engagement with symptom and lifestyle monitoring and education sections of the CareApp, as well as other sections of the CareApp (e.g., the option to share data with care network members) | Continuous data & Qualitative interview at T2 |
| Experience of ProACT | - Satisfaction with and attitudes in general toward the technology (e.g., its perceived benefits and challenges) | Continuous data & Qualitative interview at T2 |
| Self-management effectiveness | - Lifestyle management (e.g., setting and meeting activity and/or weight goals, medication taking) - Control / stabilization of symptoms (observational only) over time - No. of triage calls to participants | Continuous data & Qualitative interview at T1 & T2 |
| Role of care network | - Number and type of support relationships available in the participants’ social networks - Evolvement of the relationships over time - Impact of ProACT on the relationships / network | Qualitative interview at T1 & T2 |

**Table S3.** Care network key assessment domains and measures.

| **Care Network** | **Domain** | **Measure** | **Assessment TimePoint** |
| --- | --- | --- | --- |
| ICs, FCs / QAs, and HCPs | Demographics | Custom questionnaires | T1 |
| ICs | Carer Burden | Zarit Burden Interview [50] | T1, T2 |
| ICs, FCs / QAs, and HCPs | Satisfaction with and attitudes in general toward the technology (e.g., care network experiences with, perceptions of, and trust in the ProACT platform data) | Qualitative interview | T2 |
| ICs, FCs, and HCPs | Technology adoption | User Burden Scale [48] | T2 |
| ICs, FCs, and HCPs | ProACT evaluation and impact on care provision | Custom questionnaires | T2 |
| ICs, FCs / QAs, and HCPs | Engagement with ProACT platform | System data | Continuous |

*Note: ICs = Informal Carers; FCs = Formal Carers; QAs = Formal Care Quality Assistants; HCPs = Healthcare Professionals*
