# Supplementary material for: Scaling European Citizen Driven Transferable and Transformative Digital Health: Protocol for an Effectiveness-Implementation Hybrid Trial of a Digital Health Platform to Support Multimorbidity Self-Management
Source: JMIR Res Protoc. 2025 Nov 25;14:e74989. doi: 10.2196/74989 (PMC12690278; doi:10.2196/74989)
Supplement: Multimedia Appendix 3 [file resprot_v14i1e74989_app3.pdf]

PwM Baseline/T1 Semi-Structured Interview Guide
(Arms 1 and 2)

<u>Opening Questions</u>

1. What health conditions do you have and for approximately how long have you had them?

<u>Pre-Study Health and Well-being</u>

2. Have your health conditions affected your day-to-day life?
   Follow-Up Questions:
   a. *Have these conditions affected your day-to-day physical capacity or engagement in physical activity? If so, how?*
   b. *Have these conditions affected your day-to-day social life? If so, how?*
   c. *Have these conditions affected your day-to-day mood or mental well-being? If so, how?*
   d. *Has your life changed as the severity of your conditions has worsened? If so, how?*

3. Has having these conditions had any positive impact on your life, and if so, in what way?

4. How does managing multiple conditions compare to managing one / What is it like to manage multiple conditions?
   Prompt/Follow-Up Questions:
   a. *Can you tell me how the need to manage multiple conditions has affected your lifestyle and quality of life?*
   b. *Is there one condition that requires most attention, and if so, which one and why*
   c. *Is there one condition that is easiest to manage, and if so, which one and why?*

<u>Pre-Study Health and Well-being Self-Management</u>

5. Can you describe a typical day for you, including how you manage your conditions?
   Prompt/Follow-Up Questions:
   a. *In general, in what ways do you manage your conditions (e.g., in terms of diet, physical activity, seeking information about your conditions, and seeking support to manage them, including group-based peer support)?*
   b. *What symptoms do you monitor, how, how often, and why, if at all?*

6. Do you feel like you understand your conditions, how to manage them, and the benefits of doing so?

7. How would you describe your attitude towards managing your conditions?
   Prompt/Follow-Up Questions:
   a. *Do you feel motivated to manage your conditions, do you get tired of doing so and why?*

8. Do you feel confident to manage your conditions and why or why not?

9. Do you feel capable of managing your conditions (i.e., physical and mental capacity)? Why or why not?

10. Can you tell me about any habits you have or strategies you use to help you to manage your conditions (e.g., strategies to monitor symptoms, to adhere to prescriptions, and to maintain a healthy lifestyle; e.g., goal-setting, writing lists or maintaining a diary, placing monitoring equipment or healthy food in convenient locations, and communicating with carers or healthcare professionals)?

<u>Pre-Study Medication Management</u>

11. How do you find taking your medications?
    a. *What makes it easier to take your medications? (e.g., blister packs, using a reminder like an alert on your phone, being reminded by others, asking your pharmacist or doctor questions about the medications, taking your medication at the same time every day, pairing it with another thing that you do - e.g., before you brush your teeth, leaving your medications somewhere visible)*
    b. *What makes it harder to take your medications? (e.g., if you're away from home, if your daily routine is disrupted - e.g., on holidays or feeling unwell, when your medications change, if you cannot get to the doctor or pharmacy)*

12. Can you tell me what it's like to take different medications for different conditions?
    a. *Do you think certain medications are more important to take than others? Which ones? Why?*
    b. *Do you ever feel concerned about the medications you are prescribed to take? Why?*
    c. *How do you think your medications can help you to manage your conditions?*
    d. *Do you feel that you need to take your medications?*

<u>Pre-Study Technology Use</u>

13. Would you consider yourself to be good with technology, and why or why not?

14. Do you or have you used technology to help you to manage your conditions?
    Prompt/Follow-Up Questions:
    a. *What technology do or did you use?*
    b. *In what way do or did you use technology to manage your conditions (e.g., to monitor symptoms, manage/take your medications, facilitate lifestyle management, or facilitate communication with G.P. during a check-up)?*

15. Do you believe technology can help you and others to manage your different health conditions and your well-being, and why?
    Prompt/Follow-Up Questions:
    a. *Does or did this technology help you to manage one condition more so than another, and if so, why?*

16. Do or did you find this technology easy or challenging to use, and why?
    Prompt/Follow-Up Questions:
    a. *Did you seek and/or receive education or support to help you to use this technology?*

17. Having had an introduction to the SEURO technology (devices, iPad, CareApp) do you have any initial thoughts on it? Do you have any expectations around how easy/difficult the technology will be to use?

Pre-Study Support

18. Have you had access to any information about your conditions and how to manage them? Would you like to have such information? Why or why not?

19. Can you tell me about the support, if any, that is available to you to help you to manage your conditions (e.g., from family and friends, group-based peers, formal carers, or healthcare professionals)?
    a. Do you ask for and avail of support?
    b. Has this support evolved/changed as you have progressed from having one to multiple conditions?

20. **ARM 1 participants only:** We provided you with the option to include members of your care network in this study. Can you tell me why you decided to include/not to include members of your care network in this study (e.g., to improve communication and collaboration, your carers' understanding of your health conditions, and/or your carers' ability to provide enhanced support)?

Pre-Study Expectations

21. Can you tell me what you hope to get out of participating in this study?
    Prompt/Follow-Up Questions:
    a. *What are your expectations, if any, about the potential impact of the technology?*
    b. *ARM 1 ONLY: What are your expectations, if any about the support you may receive from your care network and the triage service?*

Closing Questions

22. Do you have any questions about the technology or the study that you would like to discuss with us?
23. Is there anything else you would like to tell us or discuss with us?
