# Supplementary material for: Scaling European Citizen Driven Transferable and Transformative Digital Health: Protocol for an Effectiveness-Implementation Hybrid Trial of a Digital Health Platform to Support Multimorbidity Self-Management
Source: JMIR Res Protoc. 2025 Nov 25;14:e74989. doi: 10.2196/74989 (PMC12690278; doi:10.2196/74989)
Supplement: Multimedia Appendix 4 [file resprot_v14i1e74989_app4.pdf]

PwM T2 Semi-Structured Interview Guide
(Arms 1 and 2)

Open the ProACT application and point to the features as needed to facilitate the discussion.

Run-through with participant what devices they had and what they were (or could have been) monitoring.

Opening Question

1. Having used ProACT for six months, what are your thoughts on it?

Engagement in Health and Wellbeing Self-Management

2. Can you describe a typical day for you during this trial, including how you managed your conditions?
    a. (Prompt) Have you used the technology—the devices and ProACT application—to manage your conditions during this trial, and if so, in what way?
    b. (Follow-Up Question) Did you use any particular devices or ProACT features more often than others? Why?
    c. (Follow-Up Question) Were there any health conditions that the devices or ProACT were more or less useful for? Why?
    d. (Follow-Up Question) Did you notice any relationship between your different symptoms or between your different symptoms and the other information collected with technology (e.g., physical activity information)?
    e. (Follow-Up Question) Thinking back to before you started the trial, how has your self-management routine changed, if at all, during this trial, and why? (e.g. symptom monitoring, medication management, physical activity, goal-setting, wellbeing)
    f. (Follow-Up Question) Did you use the technology as often as you intended? Why or why not?
    g. (Follow-Up Question) Did how you use the technology change during the course of the trial (e.g., did you use it more or less often as the trial progressed; were there any devices or ProACT features that you used more or less often as the trial progressed; e.g. symptom monitoring, medication management, physical activity monitoring, self-reporting)? Why / why not?
    h. (Follow-Up Question) Can you tell me why you continued to use the technology during the course of the trial (e.g., what motivated you to do so)?
    (OR IF APPLICABLE) … Can you tell me why you did not continue to use the technology as often you could have, and what do you think should change to persuade you to do so?
    i. (Follow-Up Question) Were there any scenarios in which the technology was more or less useful (e.g., after a GP visit or hospitalisation)?

I'm going to ask you some more detailed questions about the specific features of the ProACT application (e.g., how often you used them and your opinion of them).

3. Symptom monitoring
    a. How did you find recording and viewing your symptom information?
    b. **Trial Arm 1 PwMs**: How did you find the symptom-related alerts (i.e., the warning indicators that your health readings were outside of the recommended ranges; e.g., that your blood pressure reading was higher than recommended)?
    c. **Prompt for Trial Arm 1 PwMs:** How did you feel when you saw symptom-related alerts?
    d. **Prompt for Trial Arm 1 PwMs:** How did you respond to symptom-related alerts?
    e. Did you monitor your symptoms as often as you intended? Why or why not?

4. Medication Management
    a. How did the medication feature fit into or change your existing medication management routine?
    b. How did you find the medication prescription list feature?
    c. (Prompt) In what way did you use the prescription list feature (e.g., did you update it when your prescription changed; did you show it to your pharmacist)?
    (OR IF APPLICABLE) … Can you tell me why you did not use the prescription list feature (as often you could have), and what change, if any, might encourage you to do so?
    d. How did you find the medication tracker feature?
    e. (Prompt) In what way did you use the medication tracker (e.g., as you took your medications throughout the day, or did you use it at the end of the day)?
    (OR IF APPLICABLE) … Can you tell me why you did not use the medication tracker feature (as often you could have), and what change, if any, might encourage you to do so?
    f. Were there any scenarios in which the medication feature was more or less useful, and why (e.g., did you remember to take several medications in the morning and evening vs. a single medication at lunchtime; when your prescription changed?

5. Physical Activity Monitoring
    a. How did you find recording and viewing your physical activity information?
    (OR IF APPLICABLE) … Can you tell me why you did not use ProACT to monitor your physical activity (as often you could have), and what change, if any, might encourage you to do so?
    b. (Prompt) Were there any types of physical activity for which the technology was more or less useful (e.g., walking vs. cycling), and why?
    c. Thinking back to before the trial, how has your physical activity monitoring changed, if at all, during this trial?
    d. Thinking back to before the trial, how has your *engagement* in physical activity changed, if at all, during this trial, and why?

6. Goal-Setting (re. physical activity and weight; e.g., a goal concerning steps or distance walked per day or per week)
    a. How did you find recording and tracking physical activity goals?
    b. (Prompt) Can you explain why you chose to use this feature?
       (OR IF APPLICABLE) … Can you explain why you chose not to use this feature, and what change, if any, might encourage you to do so?
    c. How did you find recording and tracking weight-related goals?
    d. (Prompt) Can you explain why you chose to use this feature?
       (OR IF APPLICABLE) … Can you explain why you chose not to use this feature, and what change, if any, might encourage you to do so?
    e. Thinking back to before the trial, how have your goal-setting practices changed, if at all, during this trial?
    f. If you could set any other type of goals, what might they be (e.g., goals re. measuring symptoms or goals re. symptoms)?

7. Subjective Feelings (i.e., the self-report questions concerning anxiety, mood, and fatigue, and respiratory and cardiac issues)
    a. How did you find answering the self-report questions and viewing this information?
       (OR IF APPLICABLE) … Can you tell me why you did not use this feature (as often you could have), and what change, if any, might encourage you to do so?
    b. (Prompt) Were there any questions that you tended to answer and any questions that you tended to ignore—for instance, the questions concerning your anxiety or mood or the questions concerning your symptoms such as respiratory or cardiac issues—and why or why not?
    c. Did you notice any relationship between your responses and the other information collected with the technology (e.g., your symptom information or physical activity)?
    d. Did you use this feature as often as you intended, and why or why not?

8. The ProACT education section
    a. How did you find the education about your health conditions and how to manage them?
       (OR IF APPLICABLE) … Can you tell me why you did not explore this section (as often or as much you could have), and what change, if any, might encourage you to do so?
    b. How did you find the education about the technology?
       (OR IF APPLICABLE) … Can you tell me why you did not explore this section (as often or as much you could have), and what change, if any, might encourage you to do so?
    c. (Prompt) Was there any content or type of content that was more or less useful (e.g., the written material or videos; the education about your health conditions and how to manage them or about the technology and how to use it)?

d. Was there anything missing that should have been included (e.g., education about a different topic)?

I'm going to ask you some questions about the influence of the technology on your understanding of your health conditions and how to manage them, your thoughts and feelings about your health conditions, and your health-related behaviour.

9. Has the technology—the devices and ProACT—helped you to manage your conditions, and if so, in what way (or if not, why not, and what should change)?
    a. (Follow-Up Question) Has your understanding of your health conditions and how to manage them changed during the course of this trial, and if so, in what way?
    b. (Follow-Up Question) Has your attitude towards your health conditions and managing them changed during the course of this trial, and if so, in what way (e.g., has being constantly aware of your health information affected you in a positive or negative way or both [e.g., in terms of increased motivation, confidence, feelings of empowerment, and feelings of physical and mental capacity or in terms of increased anxiety, feeling tired of self-management, or feeling frustrated with technological problems])?
    c. (Prompt) You've spoken about how your self-management routine has changed since the beginning of the trial. Did the technology complement existing habits or strategies or did it fill a gap (and if so, in what way)?
    d. (Follow-Up Question) How important were these changes to you?
    e. (Follow-Up Question) How likely would these changes have been if you did not participate in the trial?
    f. (Follow-Up Question) Will you continue to manage your conditions in this way (i.e., using the devices)?
    g. (Follow-Up Question) Has the technology helped you to manage a particular condition or conditions more so than certain other conditions, and if so, in what way?

<u>During-Study Health and Wellbeing</u>

10. Have your health conditions changed during this trial (e.g., have they deteriorated, improved, or stabilized), and if so, for what reasons?
    a. (IF APPLICABLE) Has your COPD changed during this trial, and if so, for what reasons?
    b. (IF APPLICABLE) Has your asthma changed during this trial, and if so, for what reasons?
    c. (IF APPLICABLE) Has your cardiovascular health in general changed during this trial, and if so, for what reasons?
    d. (IF APPLICABLE) Has your blood pressure changed during this trial, and if so, for what reasons?
    e. (IF APPLICABLE) Has your atherosclerosis changed during this trial, and if so, for what reasons?

f. (IF APPLICABLE) Has your angina changed during this trial, and if so, for what reasons?
g. (IF APPLICABLE) Has your arrhythmia or irregular heartbeat changed during this trial, and if so, for what reasons?
h. (IF APPLICABLE) Has your heart failure changed during this trial, and if so, for what reasons?
i. (IF APPLICABLE) Has your type 1 diabetes changed during this trial, and if so, for what reasons?
j. (IF APPLICABLE) Has your type 2 diabetes changed during this trial, and if so, for what reasons?
k. (Follow-Up Question) Has your health in general changed during this trial, and if so, for what reasons?

11. Has your quality of life changed during this trial (e.g., has it deteriorated, improved, or stayed the same), and if so, for what reasons?

12. Has your mental wellbeing or mood changed during this trial, and if so, in what way?

<u>During-Study Care Network Support</u>

13. **Trial Arm 1 PwMs with participating CN members:** Members of your care network participated in this trial with you. Can you tell me about the support you received from them during this trial to help you to manage your conditions?
    a. (Follow-Up Question) Did you discuss your readings with them and for what reasons did you do so?
    b. (Follow-Up Question) Did they become more aware of your health conditions and how to provide support?
    c. (Follow-Up Question) Did conversations with your G.P. change during the course of the trial, and in what way?
    d. (Follow-Up Question) Has the support you receive from your care network members changed during the course of this trial (i.e., in comparison to before the trial), and can you describe how (e.g., in terms of conversations about your health conditions and how to manage them; in terms of their understanding of your health conditions and how to provide support; in terms of the nature of your relationship)?
    e. (Follow-Up Question) How important was your care network members' inclusion in this trial in terms of making ProACT effective (i.e., would ProACT have been as effective if only you used it)?
    f. (Prompt) Would you ask your care network members to use ProACT in future, and for what reasons?

14. **Trial Arm 1 PwMs with participating CN members:** Can you tell me about the support you received from your care network members (e.g., family members and friends or healthcare professionals), who did not formally participate in this trial, to help you to manage your conditions?

a. (Follow-Up Question) Has using the technology—the devices and ProACT application—had any impact on the support you receive (e.g., did you show your readings or prescription list to your family members and friends or your healthcare professionals, and for what reasons did you do so; did you discuss your readings with your family members and friends, and for what reasons did you do so; did your family members and friends become more aware of your health conditions and how to provide support; did conversations with your G.P. change during the course of the trial)?
b. (Follow-Up Question) Has the support you receive from your them changed during the course of this trial, and can you describe how?

15. **Trial Arm 1 PwMs without participating CN members:** You chose not to ask your care network members (e.g., family members and friends or healthcare professionals) to participate in this trial with you. Why was that?
    a. On reflection, would you make the same decision or would you ask them to participate?

16. **Trial Arm 1 PwMs without participating CN members and Trial Arm 2 PwMs:** Can you tell me about the support you received from your care network (e.g., family members and friends or healthcare professionals), if any, during this trial to help you to manage your health conditions (e.g., did they advise you on how to manage your conditions, did they do anything practical such as pick up medications or drive you to GP visits, and/or did they encourage or challenge you to manage your conditions)?
    a. (Follow-Up Question) Has using the technology—the devices and ProACT application—had any impact on the support you receive (e.g., did you show your readings or prescription list to your family members and friends or your healthcare professionals, and for what reasons did you do so; did you discuss your readings with your family members and friends, and for what reasons did you do so; did your family members and friends become more aware of your health conditions and how to provide support; did conversations with your G.P. change during the course of the trial)?
    b. (Follow-Up Question) Has the support you receive from your care network changed during the course of this trial (i.e., in comparison to before the trial), and can you describe how?

17. Has being involved in this trial changed how you seek support from your doctors (e.g., have you sought support from your doctors in circumstances that you previously would not have done so)?
    a. (If not already addressed during previous questions) Has being involved in this trial changed how you seek support from family members and friends (e.g., were you more likely to seek support; did you speak with family members and friends about your readings; did you request, in response to certain readings, that the support they provide change [e.g., in response to consistently high blood glucose readings, did you request that they cook certain meals])?

18. **Trial Arm 1 PwMs:** Can you tell me how you felt about the clinical triage service during this trial?
    a. (Prompt) What role, if any, did they play in your management of your health and wellbeing? For instance, notifying you about symptom alerts, advising you about symptom alerts, advising you about how often you should measure your symptoms, and advising you about your health in general.
    b. (Prompt) Did it have any impact on your health and wellbeing self-management, and if so, in what way?
    c. (Prompt) Did it have any impact on your health and wellbeing, and if so, in what way?
    d. (Follow-Up Question) Did it have any impact on your use of the technology, and if so, in what way?
    e. (Follow-Up Question) If you were to take part in a similar trial again, would you prefer to do so with or without the support of the triage nurses?

<u>Technology Use</u>

19. How did you find using the ProACT technology?
    a. (Prompt) Was it easy or difficult to use, and why?
    b. (Follow-Up Question) What was the biggest challenge?
    c. (Follow-Up Question) What has surprised you most about using it?
20. How did you find learning to use the technology?

<u>During-Study Project Support</u>

21. How was your experience with the helpdesk?
    a. (Follow-Up Question) Did the helpdesk help you to learn how to use the technology, and in what way?
    b. (Follow-Up Question) Did the helpdesk help you to deal with faults with the technology, and in what way?
    c. (Follow-Up Question) Did the helpdesk help you in any other ways?
    d. (Follow-Up Question) Did the helpdesk influence how you participated in the trial (e.g., did the helpdesk influence your use of the technology, staying engaged in using the technology, or your self-management activities)?

<u>Closing Questions</u>

22. What did you get out of participating in the trial?
23. Can you tell me three good things about your involvement in this trial?
24. Can you tell me three bad things about your involvement in this trial?
25. Do you think you would like to use similar technology in the future to help you manager your health and wellbeing? Why or why not?
26. Do you have questions for us that you would like us to answer?
27. Is there anything else what you would like to tell us or to discuss?

Technology-specific questions (potentially separate interview if time is tight)

During-Study Technology Use

28. How did you find using the devices, as pieces of technology?
    a. (Prompt) Were they easy or difficult to use, and why?
    b. (Follow-Up Question) What was the biggest challenge?
    c. (Follow-Up Question) What has surprised you most about using them?

29. How did you find using the ProACT application, as a piece of technology (*as before, ensure that the application is open and scroll through the different features to facilitate the discussion*)?
    a. (Prompt) Was it easy or difficult to use, and why?
    b. (Follow-Up Question) What was the biggest challenge?
    c. (Follow-Up Question) What has surprised you most about using it?

30. (If not already addressed during previous questions) How did you find navigating the ProACT application and its different features?
    a. Was it frustrating or satisfying to use, and in what way?

31. What are your thoughts on how the information was presented in ProACT?
    a. (Follow-Up Question) Was the information easy or difficult to read?
    b. (Follow-Up Question) Were the graphs easy or difficult to interpret?
    c. (Follow-Up Question) Were the graphs useful?
    d. (Follow-Up Question) Were the symbols—the green ticks, the missing information symbols, and the symptom alert symbols—useful (or noticeable and easy to interpret)?

32. (If not already addressed during previous questions) Was the technology reliable or not (e.g., in terms of connecting with the internet or recording data)?

33. (If not already addressed during previous questions) How did you find learning to use the technology?
    a. (Prompt) Were the devices and ProACT application easy to learn how to use or not?
    b. (Follow-Up Question) Was the training useful or not?
    c. (Follow-Up Question) Did you use the training manual and education videos, and if so, in what way and in what circumstances?
    d. (Prompt) Would you have liked to receive additional training or a different type of training?
    e. (Follow-Up Question) How did you feel about technology at the start of this trial, and has this changed (e.g., would you feel more confident to use technology now)?
    f. (Follow-Up Question) Would you have considered yourself to be good with technology prior to this trial, and has this changed?

g. (Follow-Up Question) Did you use technology to help you to manage your conditions prior to this trial, and how did it differ?
h. (Follow-Up Question) Did you seek support from anyone, such as family members or friends, about how to use the technology, and in what way?
