# Supplementary material for: Scaling European Citizen Driven Transferable and Transformative Digital Health: Protocol for an Effectiveness-Implementation Hybrid Trial of a Digital Health Platform to Support Multimorbidity Self-Management
Source: JMIR Res Protoc. 2025 Nov 25;14:e74989. doi: 10.2196/74989 (PMC12690278; doi:10.2196/74989)
Supplement: Multimedia Appendix 5 [file resprot_v14i1e74989_app5.pdf]

Proposal Evaluation Form

EUROPEAN COMMISSION

Horizon 2020 - Research and Innovation Framework Programme

Evaluation Summary Report - Research and innovation actions

Call: H2020-SC1-2020-Two-Stage-RTD
Type of action: RIA
Proposal number: 945449-2
Proposal acronym: SEURO
Duration (months): 36
Proposal title: Scaling EUROpean citizen driven transferable and transformative digital integrated health and social care
Activity: SC1-DTH-13-2020

N.	Proposer name	Country	Total Cost	%	Grant Requested	%
1	THE PROVOST, FELLOWS, FOUNDATION SCHOLARS & THE OTHER MEMBERS OF BOARD OF THE COLLEGE OF THE HOLY & UNDIVIDED TRINITY OF QUEEN ELIZABETH NEAR DUBLIN	IE	695,221.25	17.38%	695,221.25	17.38%
2	ASSOCIAZIONE ITALIANA PER L ASSISTENZA AGLI SPASTICI PROVINCIA DI BOLOGNA	IT	379,500	9.49%	379,500	9.49%
3	IBM IRELAND LIMITED	IE	670,000	16.75%	670,000	16.75%
4	INTERUNIVERSITAIR MICRO-ELECTRONICA CENTRUM	BE	657,157.5	16.43%	657,157.5	16.43%
5	DUNDALK INSTITUTE OF TECHNOLOGY	IE	695,423.75	17.39%	695,423.75	17.39%
6	Region Västerbotten	SE	498,750	12.47%	498,750	12.47%
7	VEREIN ZUR FOERDERUNG ASSISTIERENDER TECHNOLOGIE IN EUROPA	AT	85,000	2.13%	85,000	2.13%
8	EUROPEAN ASSOCIATION OF SERVICE PROVIDERS FOR PERSONS WITH DISABILITIES	BE	81,250	2.03%	81,250	2.03%
9	HOME INSTEAD FRANCHISING LTD	IE	40,000	1.00%	40,000	1.00%
10	Z-plus	BE	63,265	1.58%	63,265	1.58%
11	Carlow Emergency Doctors on Call LTD	IE	63,375	1.58%	63,375	1.58%
12	UMEA UNIVERSITET	SE	70,625	1.77%	70,625	1.77%
	Total:		3,999,567.5		3,999,567.5	

Abstract:
SEURO targets Europe’s 50 million multimorbid patients to proactively self-manage and offset the EU’s annual €700 billion cost of chronic disease management. SEURO aims to evaluate key factors to prepare organisations, localities and regions across the EU to scale, sustain and transfer the people-centred, digital integrated care platform ProACT (developed and successfully tested at proof of concept level under Horizon 2020, PHC-25-2015, Grant No. 689996). Research will aim to advance 3 novel implementation support tools. These consist of a transferability self-assessment tool (ProTransfer) to assess preparedness for solution transfer; a behavioural change framework (ProBCF) to optimise a solution to people’s needs; and an artificial intelligence (AI) prediction model (ProInsight) to assess the impact of a solution on health system performance. Effective Implementation Hybrid Trials (720 older adults in total, with associated care/support actors) will be carried out with ProACT within health services (Ireland, Belgium Sweden to measure platform effectiveness), with embedded process evaluations to understand the process for successful ‘real world’ implementation. Participants will have at least two of the following: cardiovascular disease (excluding Stroke), a respiratory condition and diabetes. Commercial potential will be validated during the project supported by an exploratory trial (Italy) to evaluate the impact of SEURO in facilitating a procurement process for healthcare service providers aiming to implement ProACT. SEURO engages a multidisciplinary EU consortium of 4 public and 8 private organisations (including one of the world's leading ICT companies, the largest home care provider and 2 EU service provider and technology networks). Partners have a successful collaborative track record. Together we are in a strong position to consolidate EU research assets into a new comprehensive approach for the scale-up and transfer of digital integrated care solutions.

Evaluation Summary Report

Evaluation Result

Total score: 13.50 (Threshold: 12)

Form information

SCORING

Scores must be in the range 0-5.

Interpretation of the score:

0 *The* ***proposal fails to address the criterion*** *or cannot be assessed due to missing or incomplete information.*

1 Poor. *The criterion is inadequately addressed, or there are serious inherent weaknesses.*

2 Fair. *The proposal broadly addresses the criterion, but there are significant weaknesses.*

3 Good. *The proposal addresses the criterion well, but a number of shortcomings are present.*

4 Very good. *The proposal addresses the criterion very well, but a small number of shortcomings are present.*

5 Excellent. *The proposal successfully addresses all relevant aspects of the criterion.Any shortcomings are minor.*

Criterion 1 - Excellence

Score: **4.50** (Threshold: 4/5.00 , Weight: -)

The following aspects will be taken into account, to the extent that the proposed work corresponds to the topic description in the work programme:
Clarity and pertinence of the objectives
Soundness of the concept, and credibility of the proposed methodology
Extent that proposed work is beyond the state of the art, and demonstrates innovation potential (e.g. ground-breaking objectives, novel concepts and approaches, new products, services or business and organisational models)
Appropriate consideration of interdisciplinary approaches and, where relevant, use of stakeholder knowledge and gender dimension in research and innovation content

The proposal addresses the criterion very well, but with minor shortcomings.
There is clarity and pertinence of the seven objectives. These are clearly linked to the aims, Work Packages, deliverables and milestones. SEURO makes a convincing explanation of why digital solutions are needed to provide multi-morbid person-centered integrated care. It also clearly shows the necessity of digital integration between health and social care to support people-centered care.
The proposal discusses in detail the scalability and transferability across Europe. Its aim, namely that of evaluating key factors to prepare organisations, localities and regions across Europe to scale, sustain and transfer a developed and successfully tested digital integrated care platform ProACT, is well explained.
The concept is sound with clear explanation of the three novel implementation support tools: ProTransfer, ProBCF and ProInsight, very well-linked in the systems overview and phases of the project. The proposed methodology is credible, while addressing the challenges and scope highlighted in the call and linking these to the Work Packages and objectives. There is a clear explanation of the trial design and choice of sample size based on statistical explanation, as well as clear inclusion criteria. Pilot studies are well embedded in the methodology. The proposed work goes beyond the current state-of-the art, in particular in terms of the three novel implementation support tools.
SEURO engages a multidisciplinary EU consortium of 3 public and 6 private organisations. It also emphasises a transdisciplinary approach in that it involves scientists, academics, engineers, psychologists and others with a track record in digital health and health systems research. Stakeholder groups are clearly identified and linked to the programme.
The proposal appropriately identifies and addresses issues related to access and quality of care due to COVID-19. Interoperability is mentioned a number of times, but the proposal would have benefited from more details on how interoperability will be achieved and which technology standards are to be used. Although there is consideration of the gender dimension in the research and innovation content, this is primarily reduced to sex-related differences. The specific elements of equity in access and potential utilisation among vulnerable groups (including immigrants, older cohorts and people with varying first language) could have been addressed in more detail.

Criterion 2 - Impact

Score: **4.50** (Threshold: 4/5.00 , Weight: -)

The following aspects will be taken into account:
The extent to which the outputs of the project would contribute to each of the expected impacts mentioned in the work programme under the relevant topic
Any substantial impacts not mentioned in the work programme, that would enhance innovation capacity, create new market opportunities, strengthen competitiveness and growth of companies, address issues related to climate change or the environment, or bring other important benefits for society
Quality of the proposed measures to:
- exploit and disseminate the project results (including management of IPR), and to manage research data where relevant
- communicate the project activities to different target audiences

The proposal addresses the criterion very well, but with minor shortcomings.
The SEURO consortium provides a strong overview of the expected impact of the project, with relevant and detailed deliverables. The impacts are in line in the work programme, as well as with the expected impacts as described in the call topic. The proposal has also mentioned other relevant impacts, including environmental, social, as well as COVID-19 related.
The proposal provides a robust exploitation, communication and dissemination plan, with clear goals and metrics per partner, in order to maximise the impact. The proposal explains the utilisation of trial outputs to develop a full exploitation strategy. It also envisages commercialisation of the tools, effective data management and high quality measures to exploit the project and its results.
The project has the potential to improve innovation capacity and integration of new knowledge by providing flexible, open tools for companies and service providers. The project is clearly market-oriented with the project activities geared to reach full market potential, aided by the Business Proposition and Cost-Benefit Analysis.
The barriers and obstacles identified and the measures how to address them should have been described in more depth.

Criterion 3 - Quality and efficiency of the implementation

Score: **4.50** (Threshold: 3/5.00 , Weight: -)

The following aspects will be taken into account:
Quality and effectiveness of the work plan, including extent to which the resources assigned to work packages are in line with their objectives and deliverables
Appropriateness of the management structures and procedures, including risk and innovation management
Complementarity of the participants and extent to which the consortium as a whole brings together the necessary expertise
Appropriateness of the allocation of tasks, ensuring that all participants have a valid role and adequate resources in the project to fulfil that role

The proposal addresses the criterion very well, but with minor shortcomings.
The work plan is effective and of good quality. Resources are well assigned to the work packages and are in line with the objectives and deliverables.

The management structures and procedures are appropriate including detailed and adequate risk management with identifiable risk-mitigation measures linked to the various phases of the work programme. The Innovation and Implementation Management Advisory Board will address key aspects of innovation around all Work Packages (WPs). In particular, this concerns how digital innovation can be effectively translated to the SEURO tools and the advancement of the ProACT platform linked to exploitation, communication, dissemination and commercial development. There is a dedicated WP for exploitation and economic evaluation of the project outcomes. Furthermore, activities related to market analysis, competitors, business opportunities are foreseen. All partners are expected to participate in exploitation and dissemination activities.
The consortium is made up of 12 partners, 4 academic/research partners with direct access to services for trial sites, 5 service partners, multinational organisations/companies, 3 SMEs and 2 pan-EU organisations to cover services and technology. The proposal clearly highlights the partners' expertise and complementarity, while also identifying the roles, tasks and responsibilities across the work plan. The Gantt chart also shows adequate timelines in line with tasks scheduling and deliverables/milestones.
However, minor shortcomings related to risk management were found. Although relevant legal frames and different jurisdictions in the trial countries are identified, the proposal would have benefited from a more detailed elaboration on a harmonised solution, including legal requirements for implementation and transfer. The proposed risk mitigation strategies would need more details on the risk of having a 36-months duration for what is a rather ambitious project, as well as on the persistent COVID-19 pandemic, including the concern around the need for extensive travel and associated budget.

Scope of the proposal

Status: **Yes**

Comments (in case the proposal is out of scope)

Not provided

Operational Capacity

Status: **Operational Capacity: Yes**

If No, please list the concerned partner(s), the reasons for the rejection, and the requested amount.

Not provided

Exceptional funding of third country participants/international organisations

A third country participant/international organisation not listed in General Annex A to the Main Work Programme may exceptionally receive funding if their participation is essential for carrying out the project (for instance due to outstanding expertise, access to unique know-how, access to research infrastructure, access to particular geographical environments, possibility to involve key partners in emerging markets, access to data, etc.). (For more information, see the Online Manual)

Based on the information provided in the proposal, we consider that the following participant(s)/international organisation(s) that requested funding should exceptionally be funded:
(Please list the Name and acronym of the applicant, Reasons for exceptional funding and the Requested grant amount.)

N/A

Based on the information provided in the proposal, we consider that the following participant(s)/international organisation(s) that requested funding should NOT be funded:
(Please list the Name and acronym of the applicant, Reasons for exceptional funding and the Requested grant amount.)

N/A

Use of human embryonic stem cells (hESC)

Status: **No**

If yes, please state whether the use of hESC is, or is not, in your opinion, necessary to achieve the scientific objectives of the proposal and the reasons why. Alternatively, please state if it cannot be assessed whether the use of hESC is necessary or not because of a lack of information.

Not provided

European Commission

FUNDING & TENDERS PORTAL E-RECEIPT

Digitally sealed by the European Commission
Date: 2020.10.20 08:30:47 CEST

This electronic receipt is a digitally signed version of the document submitted by your organisation. Both the content of the document and a set of metadata have been digitally sealed.

This digital signature mechanism, using a public-private key pair mechanism, uniquely binds this eReceipt to the modules of the Funding & Tenders Portal of the European Commission, to the transaction for which it was generated and ensures its full integrity. Therefore a complete digitally signed trail of the transaction is available both for your organisation and for the issuer of the eReceipt.

Any attempt to modify the content will lead to a break of the integrity of the electronic signature, which can be verified at any time by clicking on the eReceipt validation symbol.

More info about eReceipts can be found in the FAQ page of the Funding & Tenders Portal.

(https://ec.europa.eu/info/funding-tenders/opportunities/portal/screen/support/faq)
